# Preclinical Characterization of PC786, an Inhaled Small-Molecule Respiratory Syncytial Virus L Protein Polymerase Inhibitor

**DOI:** 10.1128/AAC.00737-17

**Published:** 2017-08-24

**Authors:** Matthew Coates, Daniel Brookes, Young-In Kim, Heather Allen, Euan A. F. Fordyce, Elizabeth A. Meals, Thomas Colley, Claire-Lise Ciana, Guillaume F. Parra, Vladimir Sherbukhin, Jennifer A. Stockwell, Jennifer C. Thomas, S. Fraser Hunt, Lauren Anderson-Dring, Stuart T. Onions, Lindsey Cass, Peter J. Murray, Kazuhiro Ito, Pete Strong, John P. DeVincenzo, Garth Rapeport

**Affiliations:** a Pulmocide Ltd., London, United Kingdom; b Department of Pediatrics, University of Tennessee Health Science Center, Memphis, Tennessee, USA; c Children's Foundation Research Institute at Le Bonheur Children's Hospital, Memphis, Tennessee, USA; d Sygnature Discovery Ltd., Nottingham, United Kingdom; e Department of Microbiology, Immunology and Biochemistry, University of Tennessee Health Science Center, Memphis, Tennessee, USA

**Keywords:** respiratory syncytial virus, polymerase, L protein, PC786, clinical isolate, RNA polymerases, bronchial epithelial cell, inhalation

## Abstract

Although respiratory syncytial virus (RSV) is the most common cause of lower respiratory tract infection in infants and young children, attempts to develop an effective therapy have so far proved unsuccessful. Here we report the preclinical profiles of PC786, a potent nonnucleoside RSV L protein polymerase inhibitor, designed for inhalation treatment of RSV infection. PC786 demonstrated a potent and selective antiviral activity against laboratory-adapted or clinical isolates of RSV-A (50% inhibitory concentration [IC_50_], <0.09 to 0.71 nM) and RSV-B (IC_50_, 1.3 to 50.6 nM), which were determined by inhibition of cytopathic effects in HEp-2 cells without causing detectable cytotoxicity. The underlying inhibition of virus replication was confirmed by PCR analysis. The effects of PC786 were largely unaffected by the multiplicity of infection (MOI) and were retained in the face of established RSV replication in a time-of-addition study. Persistent anti-RSV effects of PC786 were also demonstrated in human bronchial epithelial cells. *In vivo* intranasal once daily dosing with PC786 was able to reduce the virus load to undetectable levels in lung homogenates from RSV-infected mice and cotton rats. Treatment with escalating concentrations identified a dominant mutation in the L protein (Y1631H) *in vitro*. In addition, PC786 potently inhibited RSV RNA-dependent RNA polymerase (RdRp) activity in a cell-free enzyme assay and minigenome assay in HEp-2 cells (IC_50_, 2.1 and 0.5 nM, respectively). Thus, PC786 was shown to be a potent anti-RSV agent via inhibition of RdRp activity, making topical treatment with this compound a novel potential therapy for the treatment of human RSV infections.

## INTRODUCTION

Human respiratory syncytial virus (RSV) is a single-stranded, negative-sense RNA virus and a member of the family Pneumoviridae of the Mononegavirales order; it is the most common cause of childhood acute lower respiratory infection (1) and can produce severe disease in patients of any age. Infants, the elderly, as well as those having compromised cardiac, pulmonary, or immune systems are particularly vulnerable (2). In addition, RSV infection is increasingly implicated as a cause of exacerbations in patients suffering from chronic obstructive pulmonary disease (COPD) (3), asthma (4), and cystic fibrosis (5). In immunocompromised adults, approximately 50% of upper respiratory tract infections with RSV progress to pneumonia (6). RSV is the single leading cause of hospitalization of infants (7) and causes nearly 10 times more respiratory deaths than does influenza in this age group (8). RSV exists as two antigenic subgroups: A and B. RSV A viruses were formerly regarded as the subgroup of pathogens responsible for the majority of clinical disease and were reported to produce a slightly more symptomatic pathology (9, 10). However, recent studies have demonstrated that virus strains from the B subgroup also often predominate in medically important afflicted populations (11, 12). In addition, both subgroups exist in differing percentages within a single winter, and available point-of-care rapid diagnostic tests may or may not distinguish between the subgroups, thus necessitating an effective antiviral to have broad activity covering both viral subgroups.

Despite significant efforts to develop a safe and effective treatment against RSV, Food and Drug Administration (FDA)-approved drugs for this indication are limited to (aerosolized) ribavirin and the humanized monoclonal antibody palivizumab (Synagis). The latter agent targets the RSV fusion (F) protein and is restricted to prophylactic use in high-risk, pediatric patients. Furthermore, clinical variants resistant to neutralization by palivizumab have been identified (13), and no effective vaccine is currently available. The use of ribavirin is limited by its low potency against the virus, its questionable clinical benefit, and concerns over its toxicity. In recent years, new compounds intended for the treatment of RSV have been reported. These include the oral F protein inhibitors GS-5806 (phase II) (14), AK0529 (15), BTA-C585 (16), JNJ-53718678 (17, 18), and inhaled nanobody ALX-0171 (19) (phase II). In addition, ALS-8176, an orally bioavailable prodrug of the novel nucleoside RSV polymerase inhibitor ALS-8112 (phase II) (20), has entered clinical development.

The initial portal of entry by RSV is through the nose or eye rather than the mouth (21). Once established in the upper respiratory tract, the infection is able to progress readily into the lungs. The pathophysiology of RSV infection was investigated in a study of lung tissues obtained from deceased children who died within their first week of infection (22) or later (23). Samples from these individuals revealed the presence of RSV, by immunostaining, only in epithelial cells, without basal cells being affected. The specific localization of the pathogenic organism provides a particular challenge to treatment, since it requires a supereffective drug concentration to be maintained at the discrete cellular site of virus replication to be effective. Topical therapy is, therefore, an ideal approach for combating RSV infection.

RNA virus polymerase is increasingly recognized as an attractive target for development of antiviral drugs against hepatitis C virus (HCV), and RSV also expresses RNA-dependent RNA polymerase (RdRp), which transcribes and replicates its negative-sense RNA genome. The RSV large polymerase subunit (L) exhibits multiple enzymatic activities, including the capability to synthesize RNA as well as to add and methylate a cap on each of the viral mRNAs. The potential benefits of polymerase inhibition will be expected efficacy even after virus infection has already been established within cells, as opposed to treatments that only prevent infections of new cells, such as virus entry inhibitors, e.g., RSV F protein inhibitors. Recently, several compounds that inhibit RSV RdRp activity by targeting L protein have been described, such as YM-53403 (24), AZ-27 (25, 26), and Boehringer Ingelheim (BI) compound D (27). However, these agents are generally weak, have poor activity against both A and B strains, and/or have poor retention within the relevant lung tissues.

**FIG 1:**
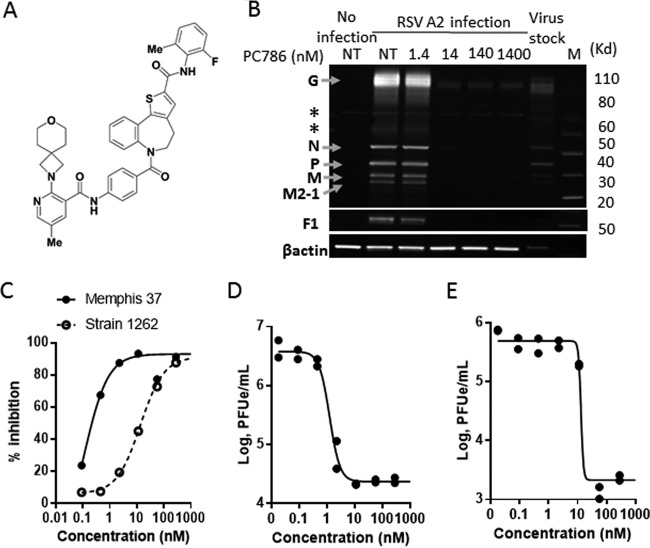
Antiviral effects of PC786. (A) Chemical structure of PC786. (B) Western blotting image demonstrating the inhibitory effects of PC786 on RSV protein expression in HEp-2 cells infected with RSV A2 for 3 days. *, nonspecific band; F1, disulfide-linked F1 subunit cleaved from RSV F0 protein precursor. NT, no PC786 treatment (0.5% DMSO only). (C) Inhibitory effects of RSV A Memphis 37- and RSV B strain 1262-induced CPE in HEp-2 cells. (D and E) PCR viral loads in the supernatants from HEp-2 cells infected with Memphis 37 (D) and strain 1262 (E) in the presence of PC786. The assay was conducted in duplicate, and individual dots show data from individual wells.

We describe here the *in vitro* and *in vivo* profiles of the novel anti-RSV therapeutic agent PC786, *N*-(2-fluoro-6-methylphenyl)-6-(4-(5-methyl-2-(7-oxa-2-azaspiro[3.5]nonan-2-yl)nicotinamido)benzoyl)-5,6-dihydro-4*H*-benzo[b]thieno[2,3-d]azepine-2-carboxamide (Fig. 1) (28), which targets the RSV L protein and is designed specifically for topical treatment by inhalation.

## RESULTS

### Antiviral activity of PC786.

The antiviral activity of PC786 (Fig. 1A) was assessed in *in vitro* cytopathic effect (CPE) assays with HEp-2 cells using laboratory-adapted RSV strains (RSV A2 and RSV B Washington [WST]), some known clinical isolates as well as low-passage-number clinical isolates with genotypic diversity, and five isolates each of RSV A and RSV B (University of Tennessee). PC786 potently inhibited CPE induced by RSV A2 and RSV B WST (Table 1). These inhibitory values were on the order of 309 to over 33,000 times more potent than those displayed by ribavirin, much more potent than those of known RSV L protein inhibitors AZ-27 and BI compound D, and equal to those of the F protein inhibitors TMC353121 and GS-5806 on RSV A2. PC786 did not affect cell viability at the concentrations up to 14 μM and consequently showed a large safety margin with respect to mammalian cell toxicity (Table 1). PC786 was found to inhibit RSV A2 protein expression in HEp-2 cells 3 days postinfection when used just after virus inoculation, and at concentrations greater than 14 nM, no or little G, N, P, M, M2-1, and F1 protein bands were visible in Western blotting (Fig. 1B). PC786 also exhibited potent inhibition of CPE induced by known RSV A clinical isolates (IC_50_, 0.14 to 3.2 nM) (Table 2).

**TABLE 1:** Comparison of *in vitro* anti-RSV activity of PC786 with those of known antiviral agents^*a*^

Compound (*n* = 3)	RSV A2		RSV B WST		Cell viability,CC_50_ (nM)
	IC_50_ (nM)	IC_90_ (nM)	IC_50_ (nM)	IC_90_ (nM)	
PC786	0.50 ± 0.0014	0.63 ± 0.035	27.3 ± 0.77	57.1 ± 3.87	>14,000
AZ-27	26.4 ± 1.16	60.0 ± 6.75	>1,580	>1,580	>15,800
BI compound D	57.6 ± 1.53	>333	47.8 ± 2.96	>1,660	600^*c*^
Ribavirin	>16,400	>16,400	10,300	>16,400	202,000 ± 80,000
ALS-8112^*b*^	455 ± 16.0	1,130 ± 77.9	647 ± 44.2	2,130 ± 363	>341,000
GS-5806	0.45 ± 0.018	0.93 ± 0.098	0.17 ± 0.043	>126	>18,800
TMC35315	0.78 ± 0.0093	1.29 ± 0.015	0.85 ± 0.017	2.63 ± 0.19	7,944 ± 2,326

a CC_50_, concentration required for 50% cytotoxicity; IC_50_ and IC_90_, concentrations required for 50 and 90% inhibition, respectively; WST, Washington.

b ALS-8112 was added 24 h prior to virus inoculation (although all others were treated simultaneously with virus infection).

c *n* = 2 (343 and 857 nM).

**TABLE 2:** Anti-RSV effects of PC786 against RSV clinical isolates in HEp-2 cells, evaluated by CPE assay

RSV A strain	Method^*a*^	IC_50_ (nM)	
		PC786	ALS-8112
1997/12-35	CPE 1	0.35	693
2001/2-20	CPE 1	0.53	768
2000/3-4	CPE 1	0.14	515
Memphis 37c	CPE 1	0.50	398
2001/3-12	CPE 2	3.2	3,200
1998/3-2	CPE 2	2.5	1,500
USU⃥v1225	CPE 2	1.3	880

a CPE 1, CPE resazurin detection in 384-well format; PC786 was used just after virus infection, and ALS-8112 was added 24 h prior to virus inoculation. CPE 2, CPE neutral red detection in 96-well format; PC786 was used just after virus infection, and ALS-8112 was added 24 h prior to virus inoculation.

PC786 also exhibited potent inhibition of CPE induced by various low-passage-number clinical isolates of RSV A (IC_50_, 0.42 nM [median]) and RSV B (IC_50_, 17.5 nM [median]) (Table 3 and Fig. 1C). The data for the individual strains are shown in Table S1 in the supplemental material. The antiviral effects of PC786 against RSV A- and RSV B-induced CPE are represented in Fig. S1A and S1B. The supernatant from RSV-infected cells was collected after the end of the CPE analysis and analyzed by reverse transcription-PCR for the RSV N gene. PC786 inhibited, in a concentration-dependent manner, N gene PCR products of the 10 clinical isolates of RSV A and RSV B, and the inhibitory activities, analyzed as IC_50_ and IC_90_ values, were consistent with those obtained from the CPE experiments (Table 3 and Fig. 1D and E; see also Table S2).

**TABLE 3:** Anti-RSV effects of PC786 against low-passage RSV clinical isolates in HEp-2 cells, evaluated in a CPE assay and by reverse transcription-PCR

RSV strain	Strain source	CPE assay result				PCR^*a*^			
		IC_50_ (nM)		IC_90_ (nM)		IC_50_ (nM)		IC_90_ (nM)	
		Median	Range	Median	Range	Median	Range	Median	Range
A2	Laboratory	0.96	NA^*b*^	1.82	NA	1.52	NA	1.69	NA
A Long	Laboratory	2.46	NA	3.25	NA	2.65	NA	3.45	NA
x5 A	Clinical	0.46	<0.09–0.71	1.77	0.57–3.77	0.54	<0.09–0.64	0.95	0.50–1.16
B 9320	Laboratory	29.1	NA	64.3	NA	30.3	NA	31	NA
x5 B	Clinical	20.2	13.4–50.6	360	27.4–821	12.1	8.10–17.7	21.7	12.3–34.0

a PCR transcripts were present in the supernatant.

b NA, not applicable.

PC786 exhibited no activity against parainfluenza virus 3 (PIV3), measles virus, influenza A virus (H1N1), rhinovirus 16, human immunodeficiency virus type 1 (HIV-1), and herpesvirus at 14 μM, suggesting a selectivity index of >33,000 in comparison to its efficacy against RSV A Long (Table 4). Although PC786 inhibited hepatitis C virus replication in the replicon assay (rather than in the CPE assay), the effective concentration (IC_50_ of 0.32 μM) was >760-fold higher than that required to inhibit RSV, and the maximum inhibition achieved was only 74% at 14 μM.

**TABLE 4:** *In vitro* antiviral effects of PC786 on selected RNA and DNA viruses

Virus	Family	Cell type	PC786				
			IC_50_ (μM)	IC_90_ or IC_95_ (μM)	CC_50_ (μM)	Safety index	Selectivity index vs RSV
RSV A long	Paramyxoviridae	HEp-2	<0.00042	<0.00042	>1.4	>3,300	
PIV3	Paramyxoviridae	LLC-MK2^*a*^ 7.1	>14	>14	>14	NA	>33,000
Measles virus	Paramyxoviridae	MRC5	>14	>14	>14	NA	>33,000
Influenza virus H1N1	Orthmyxoviridae	A549	>14	>14	>14	NA	>33,000
Hepatitis C virus	Flaviviridae	Huh-7, GT1b replicon	0.32	>14	>14	>44	>762
Rhinovirus 16	Picornaviridae	HeLa	>14	>14	>14	NA	>33,000
HIV-1	Retroviridae	CEM-SS	>14	>14	>14	NA	>33,000
Herpes simplex virus 1	Herpesviridae	Vero	>14	>14	>14	NA	>33,000

a LLC-MK2 is a rhesus monkey kidney cell line.

### Kinetics of anti-RSV activity.

**FIG 2:**
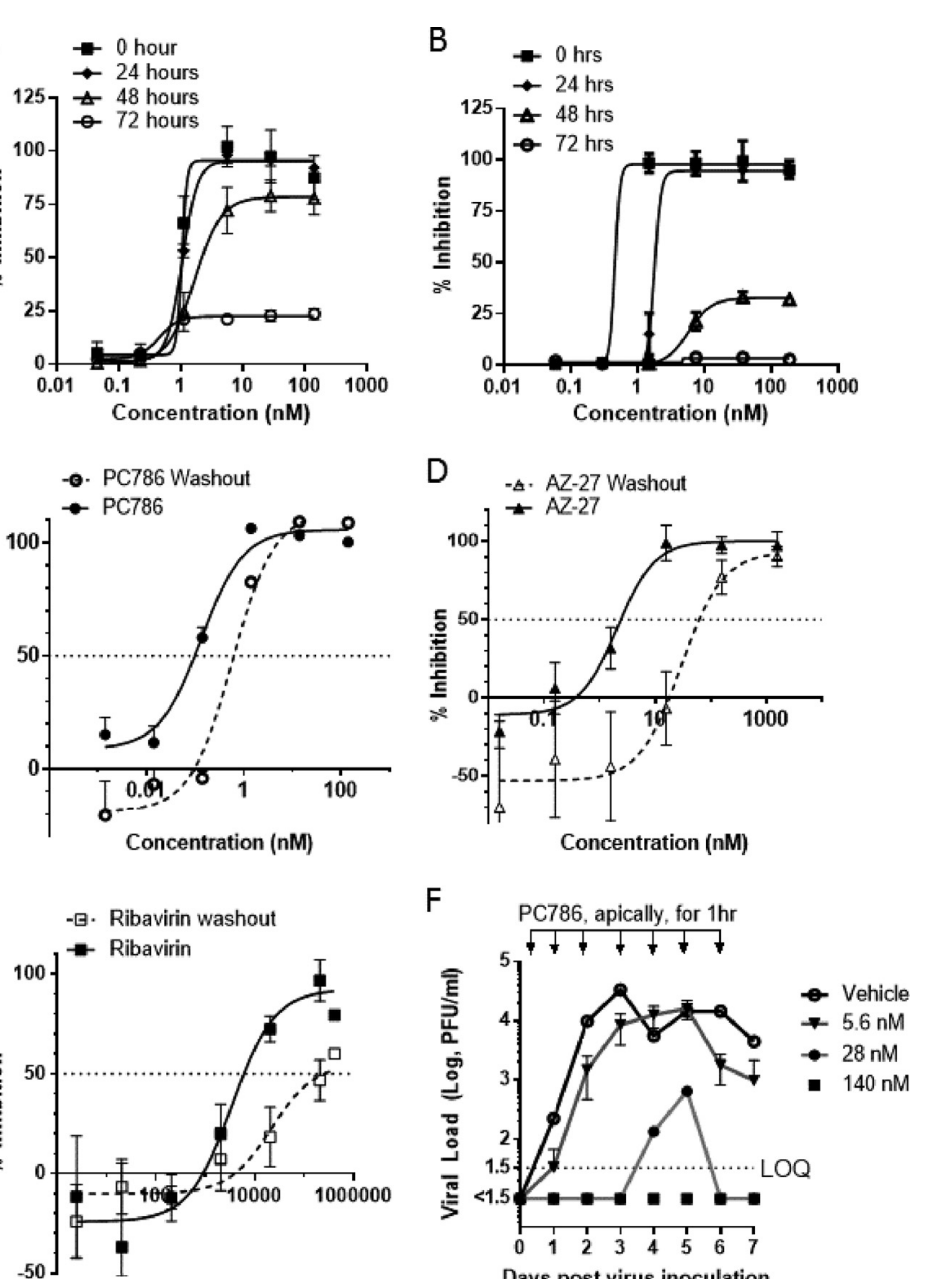
Kinetics of antiviral activity of PC786. (A) Antiviral activity of PC786 evaluated in a time-of-addition assay. PC786 was added as a single drug exposure at 0 (just after virus inoculation), 24, 48, or 72 h after infection with RSV A2, and the resulting CPE was evaluated at 144 h postinfection, showing persistence of action. (B) GS5806 data. (C to E) PC786 (C), AZ-27 (D), or ribavirin (E) was used to dose BEAS-2B cells just after virus infection (called “nonwashout”) or added for 2 h and then removed and incubated for a further 24 h before inoculation (called “washout”). Antiviral activity was assessed by detection of RSV F protein on the cell surface using enzyme-linked immunoassay. The *y* axis shows percent inhibition (mean ± SEM) versus signals from vehicle-treated virus-infected controls. (F) Time course of virus titer in apical wash from air-liquid interface-cultured differentiated human bronchial epithelial cells. After infection, apical wash with PBS was collected daily up to 7 days. After each apical wash collection, PC786 was administered for 1 h apically and then removed. The limit of quantification (LOQ) is 1.5 log PFU/ml, and virus titer lower than LOQ is shown as <1.5.

PC786 was added to HEp-2 cells at 0 (just after inoculation), 24, 48, and 72 h after inoculation with RSV A2 at multiplicities of infection (MOIs) of 0.02, 0.1, and 1, and the resulting CPE was determined at 144 h (6 days) postinoculation. The influence of PC786 on RSV A2-induced CPE was not affected by the level of infectious virus particles applied to cells (Table 5). The antiviral activity of PC786 remained even if dosing was initiated as long as 48 h after virus inoculation, and diminished thereafter (Table 5 and Fig. 2A). In contrast, the antiviral activity of the F protein inhibitor GS-5806 decreased when dosing was initiated even as short as 24 h postinoculation, and almost no antiviral activity by the F protein inhibitor was observable when initial dosing was 48 h or more after the virus inoculation (Table 5 and Fig. 2B). Additionally, this sequential diminution of the F protein inhibitor's activity was more marked at higher viral MOIs (Table 5).

**TABLE 5:** Time-of-addition study for antiviral effects of PC786 and known anti-RSV agents in RSV A infection at different MOIs in HEp-2 cells

Compound (*n* = 3)	MOI	IC_50_ (nM) at indicated treatment time (h) postinoculation				Fold increase at indicated time (h) vs time zero^*a*^		
		0	24	48	72	24	48	72
PC786	0.02	0.60	0.85	0.94	>93.4	1.4	1.6	>156
GS-5806		0.38	1.62	6.46	>188	4.3	17	>495
PC786	0.1	0.99	1.09	2.36	>140	1.1	2.4	>135
GS-5806		0.57	2.05	>188	>188	3.6	>409	>409
PC786	1.0	0.44	0.50	0.46	>140	1.1	1.0	>318
GS-5806		1.05	11.7	>188	>188	11	>181	>181

a Time zero (0 h) is just after virus inoculation.

### Persistence of action.

The persistence of action of PC786 was assessed by RSV F protein cell-based enzyme-linked immunosorbent assay (ELISA) on RSV A2-infected bronchial epithelial BEAS-2B cells. The procedure relies upon determining the effect of a 24-h washout period on the potency of PC786 compared with the compound's activity when used for treatment just after virus inoculation and left *in situ*, i.e., omitting a washout. Under this protocol, in the sustained presence of drug, the IC_50_ of PC786 against RSV A2, as measured by F protein expressed on cell surfaces, was 0.129 nM; the IC_50_ was 0.792 nM following a 24-h washout (Fig. 2C). In contrast, AZ-27's antiviral effect was diminished 35-fold from an initial IC_50_ of 3.17 nM to 106 nM after the same treatment paradigm (Fig. 2D). Ribavirin showed very weak antiviral effects (IC_50_, 5,998 nM), and this effect was diminished 42-fold (IC_50_ after washout, 252,819 nM [252 μM]) (Fig. 2E). These data indicate that short exposure to PC786 has a sustained antiviral effect compared to either ribavirin or AZ-27.

### Effects of PC786 on RSV A2 infection in ALI-cultured human airway epithelial cells.

The antiviral effect of PC786 was also evaluated using air-liquid interface (ALI)-cultured fully differentiated human primary airway epithelial cells. These cells undergo extensive mucociliary differentiation, resulting in cultures with morphological characteristics similar to those observed in the normal human respiratory epithelium. Following inoculation with a low level of RSV A2 (MOI, 0.01), the quantity of RSV in apical washes, as determined by plaque assay, in the absence of any antiviral compounds increased from day 1 to a peak at day 3 and then waned gradually and modestly up to day 7 (Fig. 2F). Treatment with PC786 to the apical surface, once daily from day 0 (1 h after virus inoculation) to day 6, induced a concentration-dependent inhibition of RSV A2 replication. This effect commenced immediately (observable within 24 h) following drug treatment on day 0 postinoculation and reduced viral quantity to below detectable limits, for the duration of the experiment, at a dose of 140 nM. When PC786 was administered at 28 nM, viral replication was not detected until day 4 and was reduced to below the level of detection once more by day 6. Viral replication was delayed using the lowest dose of PC786 (5.6 nM) and did not peak until day 5 (Fig. 2F).

### *In vivo* pharmacological effects.

Once-daily treatment with PC786, on days −1 to 3, by either intratracheal (i.t.) or intranasal (i.n.) administration, was found to inhibit viral loads in the lungs of RSV A2-infected BALB/c mice. The viral load was below the level of detection when the drug was given at 2 mg/ml (40 μg/mouse [approximately 1.6 mg/kg of body weight] for i.t. treatment, or 80 μg/mouse [approximately 3.2 mg/kg] for i.n. treatment) (Table 6).

**TABLE 6:** Effects of PC786 administered either intratracheally or intranasally on RSV load (PFU) in the lungs of RSV A2-infected mice

Treatment	No.	PC786 dose, mg/ml (approx mg/kg^*a*^)	Virus titer, log PFU/ml of lung homogenate		
			Geometric mean ± SD (*n* = 8)	Median	Interquartile range
Intratracheal dosing					
Vehicle + virus	10		3.1 ± 0.36	3.1	2.7–3.4
PC786 + virus	9	2.0 (3.2)	<0.33 (LOQ^*b*^)	<0.33 (LOQ)	NA
Intranasal dosing					
Vehicle + virus	8		3.0 ± 0.54	3.2	2.8–3.4
PC786 + virus	8	0.2 (0.19)	2.4 ± 0.59	2.5	1.9–3.0
PC786 + virus	8	2.0 (1.9)	<0.33 (LOQ)	<0.33 (LOQ)	NA

a Milligrams per kilogram are calculated at a body weight of 25 g.

b LOQ, limit of quantification.

**FIG 3:**
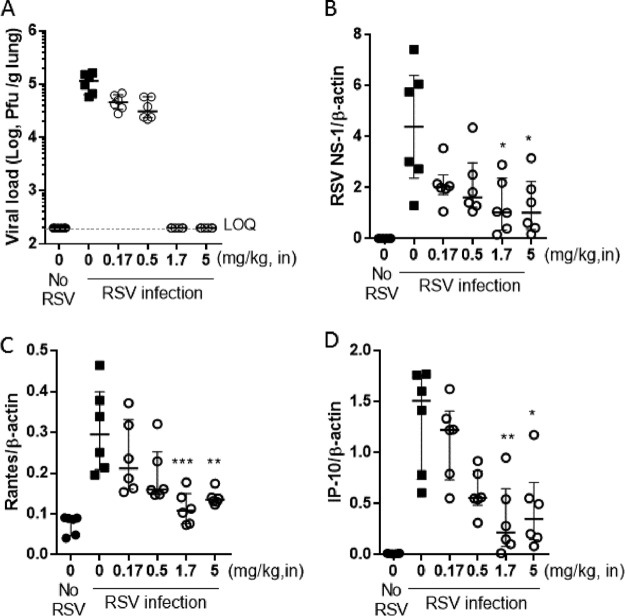
Effects of intranasal PC786 on RSV A Long-infected cotton rats. Cotton rats were inoculated intranasally with RSV A Long (1.0 × 10^5^ PFU/cotton rat), and the animals were sacrificed after 4 days. PC786 was used for treatment once daily on day 0 (4 h before infection) and then on days 1, 2, and 3. Lung homogenates were evaluated for viral load (plaque assay) (A) and PCR products (relative quantification of cDNA from lungs on day 4 after primary RSV infection) of RSV NS1 gene (B), RANTES (C), and IP-10 (D), which were normalized to that of β-actin. Individual data are plotted, and geometric means for panel A and means ± SEM for panels B, C, and D are shown. *, *P* < 0.05; **, *P* < 0.01; ***, *P* < 0.001 (versus RSV-infected control) (PC786 = 0).

A study was also conducted with cotton rats, infected intranasally with RSV A Long, in which the animals were sacrificed 4 days after the infection. Once-daily intranasal treatment on days 0 to 3 with PC786 was found to reduce the viral load in lung homogenates on day 4, and the viral load was lower than the detection limit at doses of more than 3.3 mg/ml (165 μg/cotton rat [approximately 1.7 mg/kg, i.n.]) (Fig. 3A). In addition, PC786 showed a dose-dependent inhibition of RSV NS-1 gene transcripts (Fig. 3B) and of RANTES (Fig. 3C) and IP-10 (Fig. 3D) transcripts in lung homogenates.

### Inhibitory effects of PC786 on RSV polymerase and virus gene transcription by the RSV A-derived L protein-P complex.

**FIG 4:**
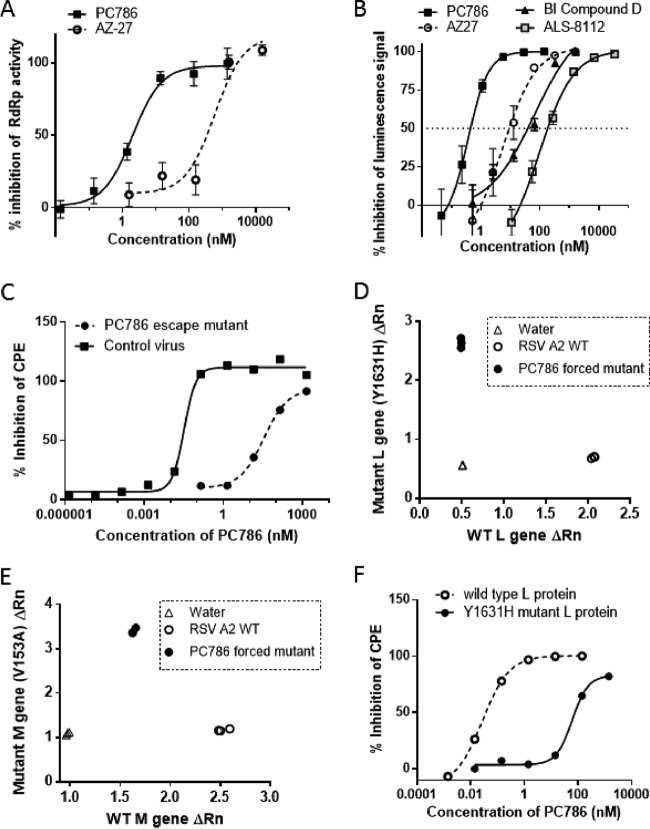
Mode of action of PC786. (A) Inhibition of PC786 on RSV RdRp activity in crude extracts from RSV L protein-P overexpressed in HEp-2 cells. (B) Inhibition of reporter fluorescent signal in RSV RdRp-driven minigenome assay. (C) Inhibitory activity of PC786 on CPE induced by control RSV A2 virus and by a PC786 escape mutant (Y1631H). (D) SNP genotype PCR analysis of viruses with wild-type and Y1631H mutant L protein. (E) SNP genotype PCR analysis of viruses with wild-type and V153A mutant M protein. (F) Minigenome analysis in HEp-2 cells transfected with wild-type and Y1631H mutant L protein plasmid.

PC786 concentration-dependently inhibited RSV RdRp activity in crude RSV A L-P complex, exhibiting an IC_50_ and IC_90_ of 2.1 nM and 21.8 nM, respectively. This profile is on the order of 75-fold to ∼163-fold more potent than that shown by AZ-27 (IC_50_, 342 nM; IC_90_, 1632 nM [Fig. 4A]). PC786 at 100 nM, which induces almost complete inhibition of RdRp activity, did not inhibit the signal from the extracts of nontreated cells, cells infected with the vaccinia virus (T7) only, or cells infected with vaccinia virus and transfected with P plasmid only (see Fig. S2).

PC786 was also found to produce potent, concentration-dependent inhibition of the expression of luciferase driven by a minigenome construct comprising the L protein, M2-1, N, and P of RSV A2 in HEp-2 cells (Fig. 4B). The maximum inhibition achieved by PC786 was 100% ± 0.09% at 140 nM, while the IC_50_ and IC_90_ were 0.476 nM and 1.54 nM, respectively. PC786 was found to be much more potent than the known RSV L protein inhibitors AZ-27 (IC_50_, 10.2 nM; IC_90_, 37.0 nM [Fig. 4B]) and BI compound D (IC_50_, 55.3 nM; IC_90_, 233 nM [Fig. 4B]). Furthermore, PC786 was also 435-fold to ∼540-fold more potent than ALS-8112 (IC_50_, 207 nM; IC_90_, 831 nM) in this minigenome assay. Only a little luciferase signal was observed in vaccinia virus only and M2-1/N/P complex only (without L protein), and PC786 at 140 nM did not show any inhibitory activities (see Fig. S3).

### Analysis of PC786 escape mutant.

RSV A2 viruses were repeatedly passaged in HEp-2 cells in the presence of increasing concentrations of PC786 (from 1.4 nM to 4,366 nM [up to 8,700-fold higher than the IC_50_] [see Table S3]). After the 6th passage, escape mutants were obtained, which exhibited IC_50_s in the CPE assays that were >650-fold weaker than those of the control virus (PC786: IC_50_ of 0.027 nM against vehicle control virus and IC_50_ of 17.9 nM against PC786 mutant virus) (Fig. 4C). Although the inhibitory activity of BI compound D on CPE was slightly reduced for the PC786 escape mutant, the same virus remained susceptible to an F protein inhibitor, GS-5806 (see Table S4). When the complete RSV genome was sequenced, the mutant at the 6th passage showed that two amino acid substitutions had occurred with high frequency; the first was tyrosine 1631 to histidine or leucine (Y1631H, Y1631L) in the RSV L gene product and the second was the change of valine 153 to alanine or methionine (V153A or V153M) in the RSV M gene product. This mutation was also confirmed by single nucleotide polymorphism (SNP) genotyping analysis using PCR (Fig. 4D and E). Both mutants arose partially in the 3rd passage, but neither was confirmed in the 2nd passage (Fig. S4). Therefore, both mutants arose at the same passage, and it appeared to happen independently rather than consequently after one mutation. Phenotypic validation of the Y1631 mutation was conducted using the RSV minigenome assay, in which the plasmid encoding the Y1631H mutated RSV L gene together with wild type N, P, and M2-1 plasmids was cotransfected into HEp-2 cells. It was found that PC786 showed a >187-fold shift in the resulting IC_50_ obtained from the minigenome assay when run against the mutated L protein (IC_50_s, 0.48 nM in wild-type L protein and 89.7 nM in mutant L protein) (Fig. 4F).

## DISCUSSION

Despite considerable effort, no safe and effective antiviral drugs to successfully treat RSV infections have emerged to date. Currently, surface proteins of RSV, such as the G and F proteins, are the principal molecular targets for the development of vaccines and small-molecule inhibitors. The RSV polymerase, which plays a central role in RSV transcription and genomic replication, is also an attractive, yet somewhat underexplored, target for therapeutic intervention. Here we report the discovery of PC786, a novel nonnucleoside small-molecule agent, and evaluate its effects. PC786 appears to inhibit the activity of the RSV RNA-dependent RNA polymerase within the large polymerase subunit (L protein). It is an *in vitro* inhibitor of virus-induced CPE in HEp-2 cells resulting from the infection with laboratory-adapted RSV A2 as well as B Washington (WST) strains. PC786 demonstrates potent anti-RSV activities against several genetically diverse low-passage-number clinical isolates in the CPE assay, although the molecule is somewhat more potent against RSV A than against B strains (Table 3). We show the antiviral activity of PC786 to be highly specific for RSV, with no inhibition observed up to 14 μM against virtually all other viruses screened (Table 4). The exception is hepatitis C virus, against which PC786 shows modest activity in the replicon assay. Furthermore, PC786 demonstrates no measurable direct cytotoxic effects in cellular activity assays conducted at concentrations up to 14 μM (Table 4) and shows no measurable inhibitory effects on human polymerase α, β, or γ on human mitochondrial polymerase up to 1 μM *in vitro* (data not shown). Thus, PC786 inhibits RSV polymerase with high specificity over polymerases expressed by other viruses and human cells.

A highly desirable feature of inhaled medicines is extended longer duration of action, thereby ensuring that therapeutic activity is maintained throughout the dosing interval. The persistence of action of PC786 was demonstrated in a washout study using BEAS2B cells (Fig. 2C). The observed persistence of effect of PC786 in the absence of RSV may be particularly valuable in the context of extended treatment dosing interval and the potential use of PC786 in prophylaxis. Once-daily treatment retains effective antiviral activity in both a human air-liquid interface (ALI)-cultured fully differentiated bronchial epithelial cells *in vitro*, as well as in mice and cotton rats *in vivo*. Of particular note is that PC786 maintains the ability to inhibit virus production irrespective of infectious particles (shown as multiplicity of infection [MOI]) and when added after peak virus production has occurred (Table 5 and Fig. 2A). In contrast, the antiviral activity of the F protein inhibitor GS-5806 was progressively diminished with increasing level of infectious particles (shown as MOI) or when administered after infection, as shown in Table 5 and Fig. 2B and previously reported (29). Because humans who are ill with RSV infection may come to medical attention at various times after the infection has started, this profile indicates that PC786 may be therapeutically advantageous for active RSV disease. Furthermore, its antiviral activity against low-passage-number human clinical isolates (Table 3) of RSV highlights these potential human therapeutic advantages.

Human RSV is able to infect and replicate in a number of animal species used for preclinical evaluation (30). Although primate species can be studied, most work of this nature is conducted with mice or cotton rats. Both standard, inbred mouse strains and cotton rats are characterized as semipermissive for the replication of human RSV, although significantly greater viral replication is seen in cotton rats than in mice. The anti-RSV activity of PC786 *in vivo* was therefore investigated in these species. Topical administration of PC786 to the lungs of mice or cotton rats subsequently challenged with RSV produced profound inhibition of virus production and exhibited a dose-dependent steep inhibition curve. In the cotton rat, PC786 was found to inhibit the RSV load, as determined by culture and PCR, in addition to gene expression of the proinflammatory molecules RANTES and IP-10 in lung homogenates. RANTES has been shown to correlate with RSV disease severity (31) and with RSV load in humans (32). Once-daily dosing indicated that treatment with PC786 resulted in sustained antiviral activity, a profile consistent with its persistence of action *in vitro* (Fig. 2C). Given that these rodent species require high inocula to establish an infection and have limited replication of RSV after infection, these models are more suitable for assessing treatments that target virus entry, rather than those reducing active intracellular replication. In fact, if administered early, several small-molecule F protein inhibitors and RSV monoclonal antibodies work well in these models (33). Therefore, we also tested the effects of PC786 on air-liquid interface-cultured fully differentiated human bronchial epithelial cells (ALI system), in which a high level of RSV replication can be sustained (Fig. 2E). Addition of RSV A2 at a low level of infectious particles (MOI, 0.01) resulted in a robust, persistent infection which generated amplified viral concentrations over 7 days. The cellular layer consisted of ciliated cells and some goblet cells, and the system shows apical shedding of progeny virions that are subsequently spread by the coordinated motion of the beating cilia, so mimicking human RSV infection (34, 35). The ALI model has been used for the evaluation of anti-RSV activities of several agents, such as RSV604 (36), ALS-8112 (37), and GS-5806 (38). In the ALI system, PC786 displayed a concentration-dependent inhibition of RSV A2 replication determined by plaque assay following daily apical exposure (mimicking a topical/aerosol delivery to the respiratory tract) commencing on day 0 and ending on day 7 postinoculation. Viral concentrations were reduced to below detectable limits for the duration of the experiment at the maximum dose investigated, 140 nM. Thus, PC786 showed a rapid onset of action (as it worked after short exposure) and produced sustained inhibition of RSV in this paradigm.

To determine the mode of action of PC786, an escape mutant was generated by passaging virus with escalating concentrations of PC786 (see Table S3). After the 6th passages with 4.4 μM PC786 (8,700× higher than the IC_50_), we were able to isolate a PC786-resistant virus that was >660-fold less sensitive to PC786 than was wild-type RSV A2 in a CPE assay (Fig. 4C). Sequencing analysis revealed that the resistant virus possessed a mutation at position 1631 in the L protein, from tyrosine to a histidine (Y1631H). This is the exact location described for mutants obtained using the other nonnucleoside polymerase inhibitors YM53403 and AZ-27 (24, 29). Phenotypic evaluation in a minigenome assay using a mutated L protein plasmid revealed that PC786 was 190-fold less active against this protein than against wild-type L protein, indicating that RSV L protein is a molecular target for PC786.

The active form of RSV polymerase is reported to be a heterodimer composed of the L and P proteins; the L protein is the catalytic subunit responsible for the RdRp function. The RSV L protein also has domains necessary for mRNA cap addition and methylation. AZ-27 was demonstrated to inhibit an early stage in mRNA transcription as well as genome replication by inhibiting initiation of RNA synthesis from the promoter. The compound did not inhibit back priming, mRNA capping, and it also did not affect association between the L and P proteins (25). AZ-27 has been also shown to inhibit synthesis of transcripts from the 3′ end of the genome to a greater extent than those from the 5′ end, indicating that it inhibits transcription initiation. AZ-27 was found to inhibit equally both mRNA transcription and genome replication in cell-based minigenome assays, indicating that it inhibits a step common to both of these RNA synthesis processes and that AZ-27 is an allosteric inhibitor of the RdRp function of RSV polymerase acting by a noncompetitive mechanism without affecting mRNA capping (39). Although a direct interaction of PC786 with a target RSV protein has not yet been demonstrated, PC786 has been shown to inhibit RSV RdRp activity in crude extracts from HEp-2 cells overexpressed with RSV L protein and P and to be highly active in a cell-based minigenome analysis (Fig. 3A and B). PC786 has therefore been shown to be an inhibitor of virus genomic RNA replication and mRNA transcription by its action on the RSV L protein. This is particularly important, as reduction in cellular expression of viral markers may be a key in rapid reduction of host immunopathology accomplishable by PC786 as opposed to nonreplication inhibitor antivirals. As PC786 induced an escape mutation at the same site as that elicited by AZ-27, the mode of action of PC786 may be similar to that of AZ-27. This allosteric inhibition by conformational change of a target protein is a common mechanism of action for nonnucleoside inhibitors of viral polymerases, such as HCV NS5B (40) and HIV reverse transcriptase (41). The L protein inhibitor BI compound D (27) was demonstrated to inhibit mRNA capping and may therefore be a different class of nonnucleoside inhibitor.

During the *in vitro* studies exposing RSV to PC786 (forced-mutation studies), a second genetic difference was observed in the M protein (change from valine 153 to alanine [V153A]), which was not reported for any anti-RSV agents, including AZ-27, BI compound D, and ALS-8112. Passage-dependent analysis revealed that the M protein mutation arose at the same time at which the L protein mutant arose, suggesting that it did not arise as a consequence of the L protein mutation (see Fig. S4). Matrix protein is known to be a key factor in virion morphogenesis (42). At an early stage of RSV replication, it is shuttled to the host cell nucleus, where it can cause modest inhibition of cellular transcription. Later, the M protein is found associated with cytoplasmic viral inclusion bodies (the site of viral RNA synthesis), in addition to the plasma membrane (the site of virion formation) (42). The M protein appears to silence viral RNA synthesis by nucleocapsids, presumably in preparation for their packaging into virions. Thus, the M protein has multiple functions which change at different stages of infection. We also conducted a minigenome assay in the presence or absence of M protein plasmid, but it did not affect the signal, suggesting that M protein did not affect promoter activation directly (Fig. S5). In addition, Do et al. have reported that the M protein is the most conserved protein between RSV A and RSV B and suggested it to be a promising target for novel anti-RSV agents (43), although genetic variations within M even occurring within a single patient with chronic RSV infection have been reported (44). It is, therefore, inferred that the principal target of PC786 inhibition arose from its action on the RSV L protein, and thereby the polymerase activity, and is probably attributable to disruption of the RSV M protein, which is involved in transcription repression, virus maturity, and budding. Presently, the significance of the M protein mutation found in association with exposure to PC786 is not fully understood, and further studies will be required.

In summary, we have identified a novel, selective, and highly potent small-molecule inhibitor that targets the replication complex of RSV and constitutes a promising candidate for the treatment of established RSV infection and disease in humans. Development to allow the study of PC786 in the clinic is under way.

## MATERIALS AND METHODS

### Materials.

PC786, GS-5806 (45), ALS-8112 (46), BI compound D (27), AZ-27 (compound 11f) (26), and AZ compound 11j (26) were prepared by Sygnature Discovery Ltd. (Nottingham, UK; final purities, >98%). RSV A2 large polymerase (L) helper plasmid (NR-36461), RSV A2 matrix 2-1 (M2-1) helper plasmid (NR-36464), RSV A2 nucleoprotein (N) helper plasmid (NR-36462), and RSV A2 phosphoprotein (P) helper plasmid (NR-36463) were obtained through BEI Resources, NIAID, NIH (Manassas, VA). Plasmids with an RSV trailer/promoter-conjugated luciferase gene (47), mutant L protein expression vector, and wild-type M protein expression vector were obtained from Creative Biogene (Shirley, NY).

### Cells and virus.

Human larynx epithelial (HEp-2) cells (ATCC CCL-23) were purchased from the American Type Culture Collection (ATCC, Manassas, VA) and maintained in 10% fetal bovine serum (FBS)-supplemented Dulbecco modified Eagle medium (DMEM) with phenol red (4190-094: Life Technologies Ltd., Paisley, UK) at 37°C and 5% CO_2_. The simian virus 40 (SV40)-immortalized human bronchial epithelial cell line BEAS-2B (ATCC) was maintained in LHC-8 medium (Invitrogen, Paisley, UK) at 37°C and 5% CO_2_. MucilAir cells were provided as 24-well plate-sized inserts by Epithelix Sàrl (Geneva, Switzerland). Twice weekly, MucilAir inserts were transferred to a new 24-well plate containing 780 μl of MucilAir culture medium (EP04MM), and once weekly the apical surface was washed once with 400 μl of phosphate-buffered saline (PBS). MucilAir cultures were incubated at 37°C and 5% CO_2_. RSV A2 (National Collection of Pathogenic Viruses [NCPV], Public Health England, Wiltshire, UK) and RSV B Washington/18537/1962 (WST; VR1580; ATCC) were propagated in HEp-2 cells. Clinical RSV A isolates 1997/12-35, 2001/2-20, and 200/3-4 were obtained from BEI Resources, NIAID, NIH (Manassas, VA), and Memphis 37c was isolated and maintained at the University of Tennessee. RSV A 2001/3-12, 1998/3-2, and USU\v1225 were sourced and used at Utah State University. Low-passage clinical isolates (RSV A Memphis 37, 1211, 689, 492, and VAN1160 and RSV B 1276, 1262, 1000, HAN1135, and PEP121) were isolated and maintained in the University of Tennessee. Vaccinia virus (ATCC; VR-2153, vTF7-3[Wr] strain) expressing bacteriophage T7 RNA polymerase was also purchased from the ATCC and used as a donor of T7 polymerase.

### CPE assay and cell viability in HEp-2 cells.

For the assay of RSV A2 and RSV B Washington (WST) strains as well as low-passage-number clinical isolates, a 96-well-format resazurin-based cytopathic effect (CPE) assay was conducted. Approximately 24 h after the cells were seeded into 96-well black plates (200 μl of 5% FBS phenol red-free DMEM/well) at a density of 3 × 10^4^/ml, the cells were infected with RSV A2, RSV A Long, or RSV A clinical strains (multiplicity of infection [MOI], 0.2), RSV A Memphis 37 (MOI, 0.6), RSV B WST (MOI, 0.2), or 9320 (VR955) or other RSV clinical strains (MOI, 1). Plain medium was also added to nontreatment wells as noninfection controls. Immediately after the infection, the compounds (except for ALS-8112) and neat dimethyl sulfoxide (DMSO; 0.5 μl/well) were added as appropriate to obtain a final concentration of 0.5% DMSO across all wells. ALS-8112 was applied 24 h before virus inoculation. The plates were incubated for 5 days for RSV A and clinical RSV B strains and 6 days for RSV B WST (37°C and 5% CO_2_). After removal of supernatant or collection of supernatant for subsequent PCR analysis, 200 μl of resazurin solution (0.0015% in PBS) was added to each well and the plates were incubated for a further 1 h at 37°C and 5% CO_2_. The fluorescence of each well (545 nm [excitation] and 590 nm [emission]) was then determined. The percent inhibition for each well was calculated against infection control and the IC_50_ and IC_90_ were calculated from the concentration-response curve generated for each test compound. For assessment of cell viability, test compounds or neat DMSO as a vehicle (1 μl) was added to each well of confluent HEp-2 cell culture in 96-well plates (200 μl of 2.5% FBS DMEM/well) and incubated for 5 or 6 days (37°C and 5% CO_2_). Cells were then incubated with resazurin solution and the fluorescence level of resorufin (metabolized materials) was determined as described above. Where appropriate, a 50% cytotoxicity concentration (CC_50_) was calculated from the concentration-response curve generated for each test compound.

For RSV A 2001/3-12, 1998/3-2, and USU⃥v1225, CPE was induced in HEp-2 cells for 7 days and the plates were then stained with neutral red dye for approximately 2 h. The intracellular incorporated dye was extracted in 50:50 Sorensen citrate buffer-ethanol and the absorbance read on a spectrophotometer. The optical density (OD) of test wells was converted to percentage of cell and virus controls. The concentration of test compound required to inhibit CPE by 50% (EC_50_) was calculated by regression analysis. The virus inocula were ∼63 50% cell culture infectious doses (CCID_50_) per well for 2001/3-12, ∼40 CCID_50_/well for 1998/3-2, and ∼32 CCID_50_/well for USU\v1225. This assay was conducted in Utah State University (Logan, UT).

For known clinical strains (RSV A 1997/12-35, 2001/2-20, 200/3-4, and Memphis 37), a 384-well-format resazurin-based CPE assay was conducted. Approximately 24 h after the cells were seeded in 384-well black plates (353962; BD Falcon, Oxford, UK) at a density of 2 × 10^4^/ml (50 μl/well in phenol red-free DMEM [31053-028; Life Technologies Ltd.] with 5% FBS, 2 mM l-glutamine [Life Technologies Ltd.], and 1 mM sodium pyruvate [Life Technologies Ltd.]), the cells were infected with RSV at an MOI of 1 (50 μl/well in phenol red-free DMEM, 2 mM l-glutamine, and 1 mM sodium pyruvate). The plain medium was also added to nontreatment wells as noninfection controls. The compounds and neat DMSO (as a vehicle) were added to appropriate wells (0.5 μl/well) using the Integra Viaflo (Integra Biosciences, Hudson, NH) to obtain a final concentration of 0.5% DMSO across all wells. The plates were incubated for 5 days (37°C and 5% CO_2_). Resazurin solution (5 μl of 0.03% solution in PBS; R7017; Sigma-Aldrich, Dorset, UK) was then added to each well using a Thermo Multidrop Combi (Thermo Fisher Scientific), and the plates were incubated for a further 6 h at 37°C and 5% CO_2_. The fluorescence of each well (545 nm [excitation] and 590 nm [emission]) was determined using a multiscanner (Clariostar; BMG, Buckinghamshire, UK).

### Time-of-addition assay and MOI dependency.

Twenty-four hours after seeding of HEp-2 cells in a 96-well plate, culture medium was aspirated from cells and replaced with 100 μl of phenol red-free DMEM containing 5% FBS. The appropriate virus concentration was diluted in 100 μl of phenol red- and serum-free DMEM to obtain final MOIs of 0.02, 0.1, and 1.0 (1,200, 6,000, and 60,000 PFU, respectively). This diluent medium was also added to noninfected wells to yield a final FBS concentration of 2.5% via an addition of 200 μl of medium to all wells. Then, 1-μl quantities of compounds and the vehicle (DMSO) were also added to obtain appropriate doses, such that the final DMSO concentration was constant across the plate at 0.5%. Compound addition was repeated on separate plates at 24, 48, or 72 h postinoculation. All plates were incubated for 6 days at 37°C and 5% CO_2_. Following the aspiration of all supernatants, 50 μl of methylene blue solution (2% formaldehyde, 10% methanol, and 8% methylene blue in 100 ml of PBS) was added to each well for 1 h. Methylene blue solution was then aspirated and cells were washed twice with 200 μl of PBS before being solubilized using 100 μl of 1% SDS solution for 1 h. The absorbance of each well was then read using a multiscanner at a wavelength of 663 nm (Clariostar; BMG, Buckinghamshire, UK). The percent inhibition for each well was calculated against vehicle-treated infected control wells.

### qPCR.

Viral RNA was extracted using EZ1 virus minikit v2.0 (Qiagen, Hilden, Germany) and EZ1 Advanced XL robot (Qiagen) per the manufacturer's protocol. Viral RNA was eluted with 60 μl of AVE elution buffer and stored at −80°C. The reverse transcription and quantitative PCR (qPCR) employed an ABI 7500 FAST real-time PCR system (Applied Biosystems International, Waltham, MA). The reverse transcription and qPCR were performed in one step by amplifying a portion of the N gene using RSV A- and B-specific primers and probes (48). The PCR mixture contained the following: 12.5 μl of 2× reaction mix (Invitrogen), 0.5 μl of SuperScript III RT/Platinum *Taq* mix (Invitrogen), 0.75 μl of ROX reference dye (Invitrogen), 0.25 μl of RNase inhibitor (Applied Biosystems; 20 U/μl), 0.045 μl (for A strains) and 0.015 μl (for B strains) of forward primers (Invitrogen; 500 μM), 0.045 μl of reverse primers (Invitrogen; 500 μM), 0.05 μl of each probe (Biosearch Technologies, Petaluma, CA; 50 μM), 10 μl of template RNA, and 0.76 μl (for A strains) and 0.79 μl (for B strains) of RNase-free water (Invitrogen). Each PCR plate contained a negative control in which RNase-free water was substituted for RNA. Each specimen was run in duplicate in 96-well plates with internal standards comprising duplicate pairs of six 10-fold dilutions of RSV RNA extracted from parallel aliquots containing a known quantity of RSV A Long (ATCC VR-26) and RSV 9320 (ATCC VR-955), as defined by and as used in the plaque assay. Results are presented as means of duplicates in log (base 10) PFU equivalents per milliliter (log PFUe/ml). The PCR employed the following thermal cycler settings: 15 min at 50°C and 2 min at 95°C, followed by 45 cycles of 15 s at 95°C and 33 s at 60°C.

### Antiviral panel screening.

The effects of PC786 against a panel of viruses were evaluated at Southern Research (Frederick, MD). The following assays were conducted: plaque reduction assay (5 days) with HEp-2 cells for RSV A (Long strain), CPE (6 days) with LLC-MK2 7.1 cells for parainfluenza virus (PIV3 C243 strain), CPE (10 days) with MRC5 cells for measles virus (Edmonton strain), CPE (5 days) with A549 cells for influenza A virus (A/PR/34 strain), CPE (3 days) with HeLa cells for human rhinovirus (HRV16 strain), CPE (5 days) with CEM-SS cells for HIV-1 (IIIB strain), CPE (4 days) with Vero cells for herpes simplex virus 1 (HF strain), and GT1b replicon luciferase assays (3 days) with Huh-7 cells for HCV. PC786 was applied 2 h before infection.

### F protein ELISA.

Approximately 72 h after BEAS-2B cells were seeded in 96-well plates (at 4 × 10^4^/ml; 100 μl/well), the medium in the plates was removed and replaced with 200 μl of fresh LHC-8 medium. In a washout arm, compounds and DMSO as the vehicle were added to appropriate wells (1 μl/well) to obtain a final concentration of 0.5% DMSO across the plate. The plates were incubated at 37°C and 5% CO_2_ for 2 h, the medium was removed and replaced with 100 μl of fresh LHC-8 medium, and the plates were subsequently incubated at 37°C and 5% CO_2_ for approximately 24 h. The cells were then infected with RSV A2 at an MOI of 0.1 in LHC-8 medium (100 μl/well) and incubated for 3 days (37°C and 5% CO_2_). For nonwashout plates, the compounds and DMSO (1 μl/well) were applied immediately after infection. On day 3 postinfection, supernatant was aspirated and the cells were fixed with 4% formaldehyde (100 μl in PBS solution) for 20 min at room temperature (RT), washed three times with wash buffer (WB) (200 μl; PBS containing 0.05% Tween 20), and incubated with blocking buffer (100 μl; 5% bovine serum albumin [BSA] in wash buffer) for 1 h. Cells were then washed with WB three times (200 μl) and incubated overnight at 4°C with anti-RSV F fusion protein antibody2F7 (50 μl/well; 1:2,000 dilution; ab43812; Abcam plc, Cambridge, UK). After a washing, cells were incubated with a horseradish peroxidase (HRP)-conjugated anti-mouse IgG antibody (50 μl/well; 1:2,000, Abcam) for 1 h. Tetramethylbenzidine (TMB) substrate (50 μl) was then added after two washings with WB and one with PBS, and the reaction was stopped by the addition of aqueous sulfuric acid (2 N; 50 μl/well). The resultant signal was determined colorimetrically (OD at 450 nm [OD_450_] with a reference wavelength of 655 nm) in a microplate reader (Multiskan FC; Thermo Fisher Scientific). Cells were then washed, and 0.5% crystal violet solution (50 μl/ml) was applied for 30 min. After two washings with PBS (200 μl), 1% SDS (100 μl) was added to each well, and the plates were shaken lightly for 1 h prior to reading of the absorbance at 595 nm as an indicator of cell number in each well. The measured F protein signals (OD_450_ − OD_655_ readings) were corrected for cell number (OD_595_ readings) by dividing the OD_450–655_ by the OD_595_ readings.

### Virus infection on ALI-cultured bronchial epithelial cells and plaque assay.

On the day of infection (day 0), the apical surface of each insert was washed once with 300 μl of sterile PBS and the inserts were then transferred to new 24-well plates containing 780 μl of fresh MucilAir culture medium (Epithelix Sàrl). RSV A2 was diluted in MucilAir culture medium to obtain a final inoculation concentration of 2,000 PFU in 50 μl (an approximate MOI of 0.01) and incubated onto cells for 1 h at 37°C and 5% CO_2_. The virus inoculum was removed with a pipette and inserts were washed twice with 300 μl of sterile PBS. A day 0 sample was harvested by adding 300 μl of sterile PBS to the apical surface of each well for 5 min. The 300-μl sample was then removed and transferred to 0.5-ml tubes containing 100 μl of 50% sucrose dissolved in PBS, and the tubes were stored at −80°C. This harvesting procedure was repeated daily until day 7. PC786 was dosed apically onto MucilAir inserts. PC786 solution diluted with PBS at 1 in 200 (50 μl; 0.5% DMSO in final solution) was applied to the apical surface and incubated at 37°C and 5% CO_2_ for 1 h before being removed. The dosing regimen was performed daily on day 0 to day 6 following each sample collection. On day 5, the basal medium was removed from every well and replenished with fresh MucilAir culture medium as a necessary maintenance step for ALI culture cells. Virus titer in the apical wash samples was determined by plaque assay. HEp-2 cells were grown in 24-well plates (Corning, Corning, NY) for 48 h prior to infection in 10% FBS DMEM until they attained 100% confluence. Collected samples (as described above) were thawed at RT and 10-fold serial dilutions were prepared in serum-free DMEM. The growth medium from HEp-2 cells was aspirated and replaced with 300 μl of serially diluted virus collections, and infection was allowed to proceed at 37°C and 5% CO_2_ for 4 h. The infectious media were aspirated and replaced with 500 μl of plaque assay overlay (1% methylcellulose in minimal essential medium [MEM]–2% fetal calf serum [FCS]), and samples were left for 7 days at 37°C and 5% CO_2_. Cells were fixed with ice-cold methanol for 10 min before the methanol was removed and the cells were washed twice with sterile PBS. Anti-RSV F protein antibody 2F7 (Abcam) was diluted to a 1:150 concentration in blocking buffer (5% powdered milk [Marvel, Premier Food, Hertfordshire, UK] in 0.05% PBS–Tween 20) and 150 μl was added to cells for 2 h at RT with shaking. Cells were washed twice with PBS before 150 μl of secondary antibody (goat anti-mouse HRP conjugate [Dako Ltd., Ely, UK; P044701-2]), diluted 1:400 in blocking buffer, was added to cells for 1 h at RT, with shaking. The secondary antibody solution was removed and cells were washed twice with PBS before the metal-enhanced development substrate 3,3′,4,4′ diaminobenzidine tetrahydrochloride (DAB) was prepared in ultrapure water (according to the manufacturer's instructions). Each well received 150 μl of development substrate (sigmaFAST D0426; Sigma-Aldrich) until plaques were visible. Plaques were counted by eye and confirmed using light microscopy.

### Western blotting.

HEp-2 cells were seeded in 6-well plates at a density of 3 × 10^5^/well (2 ml/well) 24 h before experimentation and were then treated with compounds (10 μl/well), simultaneously infected with RSV A2 (MOI, 1), and incubated for 72 h. Cells were washed with PBS and collected by scraping in 200 μl of PBS with protease and phosphatase inhibitor cocktail (PPI; Sigma-Aldrich; MSSAFE-1VL 1× in PBS), followed by two further washes with 200 μl of PBS, and the cell suspension was centrifuged to pellet. Radioimmunoprecipitation assay (RIPA) buffer (50 μl) containing 50 mM Tris-HCl (pH 7.4), 150 mM NaCl, 1% (vol/vol) NP-40, 0.25% (wt/vol) sodium deoxycholate, and PPI was applied to the cell pellet, which was vortexed and incubated for 30 min, on ice with further cell disruptions every 10 min. The protein concentration of the supernatant after centrifugation was determined by Bradford assay. The lysate containing 30 μg of protein was separated by electrophoresis on a 4 to 12% gradient bis-Tris polyacrylamide gel (Life Technologies; NP0335BOX) and then transferred to a nitrocellulose membrane using the iBlot gel transfer device (Life Technologies). The membrane was blocked with blocking buffer (TBS/T; 20 mM TBS and 0.1% Tween 20 with 5% skim milk) for 1 h at RT and incubated with anti-RSV F protein antibody (Abcam ab43812; 1:1,000) overnight at 4°C for F protein detection. The membrane was then washed three times with TBS/T and incubated with secondary antibody (goat anti-mouse HRP conjugate; Sigma-Aldrich; P0447) for 2 h at RT. After three washes with TBS/T, equal volumes of ECL Prime Western blotting detection reagent (Amersham 12994780; GE Healthcare Life Sciences, Buckinghamshire, UK) were added to the blot and incubated at RT for 3 min. The immunoreactive bands were visualized in an imaging scanner (Syngene G; box, software; GeneSys, Syngene UK, Cambridge, UK). Membranes was also reprobed with anti-RSV antibody (Virostat [Westbrook, MA] 0601; 1:1,000; overnight at 4°C) and with the secondary antibody (rabbit anti-goat HRP conjugate; Sigma-Aldrich; P0449; 2 h at RT) to detect other RSV proteins or with anti-β-actin antibody (Abcam ab6276; 1:5,000; 2 h at RT; 2° goat anti-mouse HRP conjugate; Sigma-Aldrich; P0447; 2 h at RT).

### RdRp activity assay using HEp-2 cell extract.

Inhibition of RSV RdRp activity was assessed by Cy5-CTP/ATP incorporation into the primer of the promoter region, in a modified primer extension assay previously reported (39). Approximately 70% confluent HEp-2 cells in a T-75 flask were infected with vaccinia virus (expressing T7 RNA polymerase) at an MOI of 0.1 and incubated at 37°C and 5% CO_2_ for 1 h, and then virus was removed. Plasmids (1 μg of P- and 0.5 μg of L protein-encoding plasmid) in Lipofectamine 2000 (ratio of transfection reagent to DNA, 3 μl to 1 μg) diluted in 5 ml of Opti-MEM were transfected and left for 2 h at 37°C and 5% CO_2_ before a further 12 ml of DMEM containing 2% FBS was added to the cells. Cells were collected at 48 h posttransfection, and crude L protein-P complex was extracted as an S3 fraction as previously described (49). Compound or DMSO solution (0.5 μl) was applied to a buffer (46.5 μl) containing 20 mM Tris-HCl (pH 7.5), 10 mM KCl, 6 mM MgCl_2_, 2 mM dithiothreitol (DTT), 0.01% Triton X-100, 3% DMSO, 5 U/ml of RNase inhibitor, 10 μM GTP, 10 μM UTP, 10 μM Cy5-conjugated ATP (catalog number NEL597001EA; PerkinElmer, Waltham, MA), 10 μM Cy5-conjugated CTP (catalog number NEL581001EA; PerkinElmer), 200 nM RNA (5′-UUUGUUCGCGU; Sigma-Aldrich), and 4 μM 5′ biotin-conjugated RNA primer (5′-biotin-ACGC; Sigma-Aldrich) and then mixed and incubated in the dark for 5 min at RT. The primer extension reaction was started by adding crude RSV polymerase L protein-P complex (20 μg of protein in 3 μl) to the reaction buffer prepared as described above, the buffer was incubated at 30°C for 120 min, and the reaction was stopped by the addition of 10 μl of 50 mM EDTA. The solution was transferred to a streptavidin-coated 96-well black plate (Pierce 15119; Thermo Fisher Scientific), incubated for 45 min in the dark, and then washed twice with 20 mM Tris-HCl containing 0.1% Tween 20 (pH 7.5). Diethyl pyrocarbonate (DEPC)-treated H_2_O (60 μl) was added to the plate to aid detection, and the fluorescence level of Cy5 was measured using the Clariostar reader (BMG Labtech, Buckinghamshire, UK), with the following wavelengths (bandwidth): excitation, 610 (30) nm; emission, 675 (50) nm; and dichroic, 637.5 nm.

### Minigenome analysis.

The RSV trailer/promoter-conjugated luciferase plasmid reported previously (47) was manufactured by Creative Biogene (NY). Briefly, four overlapping oligonucleotides were annealed to form a 238-bp DNA fragment containing a terminal BamHI site, the upstream 32-nucleotide (nt) nonstructural protein 1 (NS1) nontranslated region, the 10-nt RSV NS1 gene start signal, a 44-nt RSV leader sequence, a 94-bp hammerhead ribozyme, a 47-bp T7 terminator, and a NotI-compatible end. A 191-bp DNA fragment was synthesized *in vitro*, containing a terminal HindIII site, a 155-nt RSV trailer sequence, the 12-nt RSV L gene end sequence, a 12-nt nontranslated region of RSV L, and an XhoI site (Integrated DNA Technologies, Skokie, IL). These two fragments were ligated along with a BamHI/XhoI fragment of firefly luciferase cDNA (pGEM-luc; Promega Corp., Fitchburg, WI) into the NotI and HindIII sites of pcDNA3.1 such that an antisense copy of luciferase flanked by RSV leader and trailer regulatory elements was produced by T7 polymerase transcription. On the day of transfection, growth medium was removed from HEp-2 cells and replaced with 50 μl of serum-free DMEM containing vaccinia virus (expressing T7 polymerase) at an MOI of 0.1, with one row of cells receiving serum-free medium only as uninfected controls. Cells were incubated at 37°C and 5% CO_2_ for 1 h, during which time plasmids were prepared for transfection using Lipofectamine 2000 as per the manufacturer's instructions. A master mix was prepared allowing for 0.00625 μg of M2-1-, N-, P-, and Luc-encoding plasmids and 0.003215 μg of L protein-encoding plasmid per 60 μl of Opti-MEM to be administered per well, with a final ratio of Lipofectamine 2000 to DNA of 3 μl to 1 μg. Medium containing vaccinia virus was removed and the transfection reagents were added to the cells and left for 2 h at 37°C and 5% CO_2_ before a further 140 μl of DMEM containing 2% FBS was added to the cells. Compounds were administered in 1-μl volumes at this time point and cells were further incubated for 48 h at 37°C and 5% CO_2_. Luminescence was then detected using the luciferase assay system (Promega Corp.) as follows. Cells were lysed in 100 μl of 1× cell culture lysis reagent (Promega Corp.), and 20 μl of each well was transferred to a black flat-bottomed 96-well plate (Thermo Fisher Scientific, Waltham, MA). Each well was injected with 100 μl of luciferase assay reagent, and luminescence was detected for 5 s by a multiscanner (Clariostar). A single nucleotide change, T13388C (full genome numbering), was engineered into the L protein sequence to generate the Y1631H mutation in the L protein (Creative Biogene), and a pcDNA 3.1(+) vector harboring mutated L protein was also used for a minigenome analysis.

### Mutation induction.

HEp-2 cells, maintained in DMEM with phenol red supplemented with 10% FBS at 37°C and 5% CO_2_, were subcultured twice a week. Approximately 72 h prior to the beginning of the experiment, cells were seeded in T-75 flasks in 10% FBS DMEM to be fully confluent on the day of infection. RSV A2 (MOI of 0.2) was added to T-75 flasks of HEp-2 cells and incubated for 2 h at 37°C and 5% CO_2_, after which the virus was removed and replaced with 12 ml of the medium containing the appropriate compound dilutions. The dosing regimen used is shown in Table S3 in the supplemental material. The flasks were incubated for 4 to 8 days at 37°C and 5% CO_2_ until a cytopathic effect was seen in the flasks. The medium containing floating cells and cell debris (due to CPE) was collected and centrifuged at 1,200 rpm for 10 min. Cell-free supernatant was collected into sucrose solution (a final sucrose concentration of 12.5% [wt/vol]) and stored at −80°C. For the next passage, the virus stock from the previous passage (1 ml) was applied to T-75 flask containing confluent HEp-2 cells. This procedure was then repeated. The viral titer of all virus samples was determined by plaque assay. Collected samples were thawed at RT and 10-fold serial dilutions were prepared in serum-free DMEM. The growth medium (10% FBS DMEM) from HEp-2 cells grown in 24-well plates was aspirated and replaced with 300 μl of serially diluted virus collections, and the plates were left to allow infection at 37°C and 5% CO_2_ for 4 h. The infectious medium was aspirated and replaced with 1 ml of plaque assay overlay (0.3% Avicel RC-591 [FMC Biopolymer UK, Girvan, Scotland] in MEM supplemented with a final concentration of 2% FBS) and the plates were left for 7 days at 37°C and 5% CO_2_. Cells were fixed with ice-cold methanol for 10 min and then subjected to staining with 200 μl of 0.1% crystal violet solution, in distilled water, for 1 h. The crystal violet solution was removed and the cells were rinsed with water before plaques were counted and virus titer was enumerated.

### Virus full-genome sequence.

Viral RNA was extracted from serially passaged virus stocks using a QIAamp viral RNA minikit (Qiagen, Hilden, Germany) according to the manufacturer's instructions. Approximately 55 μl of extracted RNA was then dispatched to GENEWIZ Inc. (South Plainfield, NJ) for full-genome sequencing using the MiSeq technique, and single nucleotide polymorphisms (SNPs) were identified against a template RSV A2 sequence (GenBank accession number M74568.1). SNPs that appeared with a frequency of more than 20% in a routinely used laboratory strain of RSV were identified as drift mutations that emerged in-house during routine passage at Pulmocide Ltd. These nucleotide changes were used to amend the submitted RSV A2 sequence template so that a direct comparison could be made against in-house RSV A2 isolates as opposed to the published sequence mentioned above. SNPs which appeared with a frequency of over 10%, or that matched previously reported mutation sites, were analyzed to see if they elicited a codon change. Silent mutations were excluded from the report.

### PCR genotype assay.

To confirm the genotype of the compound-treated viruses, a TaqMan SNP genotype assay to detect mutant (Y1631H) and wild-type virions was designed. Viral RNA was extracted from the appropriate virus samples by MagMAX Express, and these were used to produce cDNA by reverse transcription using Invitrogen SuperScript VILO MasterMix (SuperScript; Life Technologies) as per the manufacturer's instructions. The TaqMan SNP genotyping PCR assay was run using TaqMan genotyping master mix (catalog number 4371355; Life Technologies) in the StepOne Plus PCR instrument, with enzyme activation at 95°C for 10 min, followed by 40 cycles of 15 s at 95°C and then 60 s at 60°C, with fluorescence measured. The data were analyzed using StepOne software V2.3 to determine which allele of the target gene was present in each solution. The primers used for this SNP assay were as follows: forward (GAAGACACTCAACCCTACACATGAT), reverse (ACACTGATGGATCTTAGGTATGTTGGT), reporter VIC conjugated (ATTTGAAAACATAGTAACATCA), and reporter 6-carboxyfluorescein (FAM) conjugated (AAAACATAGCAACATCA) for M protein and forward (ACAGTTTGCCCTTGGGTTGTTAA), reverse (TCCCATTCTAACAAGATCTATATAAGTTAATATTGCTTTC), reporter VIC conjugated (TGTTGGATGATAATCTATG), and reporter FAM conjugated (TTGGATGATGATCTATG) for L protein.

### *In vivo* RSV infection.

BALB/c mice (male, 20 to 30 g) were inoculated intranasally on day 0 with RSV A2 (0.65 × 10^5^ PFU/mouse) in Pneumolabs UK. Animals were sacrificed 4 days after the inoculation, and the lungs were collected for preparation of lung homogenate. PC786 was prepared as suspension in 10% DMSO–90% isotonic saline and delivered using an intratracheal installation of 20 μl/mouse or intranasal installation of 40 μl/mouse at a dose of 0.2 or 2 mg/ml on day −1, again on day 0 (1 h before inoculation), and then once daily on days 1, 2, and 3 postinoculation. Cotton rats (males, 6 to 8 weeks old; body weight, approximately 100 g) were infected intranasally with RSV A Long (1.0 × 10^5^ PFU/cotton rat) on day 0 in Sigmovir Biosystems Inc. (Rockville, MD). Animals were sacrificed 4 days after the inoculation of virus, and lungs were collected for preparation of lung homogenate. PC786 was prepared as a suspension in 10% DMSO–90% isotonic saline and delivered intranasally by installation of 50 μl/cotton rat at a dose of 0.33, 1.0, 3.3, or 10 mg/ml on day 0 (4 h before infection) and then once daily on days 1, 2, and 3 after inoculation. Virus load in all animal lung homogenates was determined by plaque assay using HEp-2 cells. In both animals, lung homogenates from uninfected animals were used for negative control. In both studies, the dose unit of milligrams per kilogram was also calculated based on approximate average body weight on day 0 (mice, 25 g, and cotton rats, 100 g).

### Statistical analysis.

Results are represented as means ± standard errors of the means. IC_50_, IC_90_, and CC_50_ were calculated using Dotmatics software (Dotmatics Ltd., Hertfordshire, UK) or GraphPad Prism (GraphPad Software Inc., La Jolla, CA). The safety index was calculated as the ratio of the CC_50_ and IC_50_. Multiple comparison was performed by analysis of variance (ANOVA) followed by Dunnett's multiple-comparison test performed using the PRISM 6 software program. If no significance was achieved using ANOVA, nonparametric Kruskal-Wallis analysis followed by Dunn's multiple-comparison test was conducted. The comparison between two groups was performed by unpaired *t* test with Welch's correction or the Mann-Whitney test. Statistical significance was defined as a *P* value of <0.05.

## Supplementary Material

Supplemental material

## References

[1] Nair H, Nokes DJ, Gessner BD, Dherani M, Madhi SA, Singleton RJ, O'Brien KL, Roca A, Wright PF, Bruce N, Chandran A, Theodoratou E, Sutanto A, Sedyaningsih ER, Ngama M, Munywoki PK, Kartasasmita C, Simoes EA, Rudan I, Weber MW, Campbell H 2010 Global burden of acute lower respiratory infections due to respiratory syncytial virus in young children: a systematic review and meta-analysis. Lancet 375:1545–1555. doi:10.1016/S0140-6736(10)60206-1. 20399493 PMC2864404

[2] Walsh EE, Falsey AR 2012 Respiratory syncytial virus infection in adult populations. Infect Disord Drug Targets 12:98–102. doi:10.2174/187152612800100116. 22335500

[3] Mehta J, Walsh EE, Mahadevia PJ, Falsey AR 2013 Risk factors for respiratory syncytial virus illness among patients with chronic obstructive pulmonary disease. COPD 10:293–299. doi:10.3109/15412555.2012.744741. 23536980

[4] Darveaux JI, Lemanske RF Jr 2014 Infection-related asthma. J Allergy Clin Immunol Pract 2:658–663. doi:10.1016/j.jaip.2014.09.011. 25439354 PMC5516525

[5] Abman SH, Ogle JW, Butler-Simon N, Rumack CM, Accurso FJ 1988 Role of respiratory syncytial virus in early hospitalizations for respiratory distress of young infants with cystic fibrosis. J Pediatr 113:826–830. doi:10.1016/S0022-3476(88)80008-8. 3183835

[6] Abdallah A, Rowland KE, Schepetiuk SK, To LB, Bardy P 2003 An outbreak of respiratory syncytial virus infection in a bone marrow transplant unit: effect on engraftment and outcome of pneumonia without specific antiviral treatment. Bone Marrow Transplant 32:195–203. doi:10.1038/sj.bmt.1704116. 12838285

[7] Leader S, Kohlhase K 2002 Respiratory syncytial virus-coded pediatric hospitalizations, 1997 to 1999. Pediatr Infect Dis J 21:629–632. doi:10.1097/00006454-200207000-00005. 12237593

[8] Thompson WW, Shay DK, Weintraub E, Brammer L, Cox N, Anderson LJ, Fukuda K 2003 Mortality associated with influenza and respiratory syncytial virus in the United States. JAMA 289:179–186. doi:10.1001/jama.289.2.179. 12517228

[9] Melero JA, Moore ML 2013 Influence of respiratory syncytial virus strain differences on pathogenesis and immunity. Curr Top Microbiol Immunol 372:59–82. 24362684 10.1007/978-3-642-38919-1_3 PMC4880365

[10] Panayiotou C, Richter J, Koliou M, Kalogirou N, Georgiou E, Christodoulou C 2014 Epidemiology of respiratory syncytial virus in children in Cyprus during three consecutive winter seasons (2010–2013): age distribution, seasonality and association between prevalent genotypes and disease severity. Epidemiol Infect 142:2406–2411. doi:10.1017/S0950268814000028. 24476750 PMC9151279

[11] Ren L, Xiao Q, Zhou L, Xia Q, Liu E 2015 Molecular characterization of human respiratory syncytial virus subtype B: a novel genotype of subtype B circulating in China. J Med Virol 87:1–9. doi:10.1002/jmv.23960. 24910250

[12] Auksornkitti V, Kamprasert N, Thongkomplew S, Suwannakarn K, Theamboonlers A, Samransamruajkij R, Poovorawan Y 2014 Molecular characterization of human respiratory syncytial virus, 2010-2011: identification of genotype ON1 and a new subgroup B genotype in Thailand. Arch Virol 159:499–507. doi:10.1007/s00705-013-1773-9. 24068580

[13] Zhu Q, McAuliffe JM, Patel NK, Palmer-Hill FJ, Yang CF, Liang B, Su L, Zhu W, Wachter L, Wilson S, MacGill RS, Krishnan S, McCarthy MP, Losonsky GA, Suzich JA 2011 Analysis of respiratory syncytial virus preclinical and clinical variants resistant to neutralization by monoclonal antibodies palivizumab and/or motavizumab. J Infect Dis 203:674–682. doi:10.1093/infdis/jiq100. 21208913 PMC3072724

[14] DeVincenzo JP, Whitley RJ, Mackman RL, Scaglioni-Weinlich C, Harrison L, Farrell E, McBride S, Lambkin-Williams R, Jordan R, Xin Y, Ramanathan S, O'Riordan T, Lewis SA, Li X, Toback SL, Lin SL, Chien JW 2014 Oral GS-5806 activity in a respiratory syncytial virus challenge study. N Engl J Med 371:711–722. doi:10.1056/NEJMoa1401184. 25140957

[15] Blair W, Cox C 2016 Current landscape of antiviral drug discovery. F1000Res 5(F1000 Faculty Rev):202 https://f1000research.com/articles/5-202/v1. 10.12688/f1000research.7665.1 PMC4765712 26962437

[16] ClinicalTrials.gov. 2016 Safety, efficacy and pharmacokinetics of BTA-C585 in a RSV viral challenge study (NCT02718937). https://clinicaltrials.gov/ct2/show/NCT02718937.

[17] Israel S, Rusch S, DeVincenzo J, Boyers A, Fok-Seang J, Huntjens D, Lounis N, Marien K, Stevens M, Verloes R 2016 Effect of oral JNJ-53718678 (JNJ-678) on disease severity in healthy adult volunteers experimentally inoculated with live respiratory syncytial virus (RSV): a placebo-controlled challenge study. Open Forum Infect Dis 3:650. doi:10.1093/ofid/ofw172.513.

[18] ClinicalTrials.gov. 2016 Study to evaluate antiviral activity, safety, and pharmacokinetics of repeated doses of orally administered JNJ 53718678 against respiratory syncytial virus infection (NCT02387606). https://www.clinicaltrials.gov/ct2/show/NCT02387606.

[19] Detalle L, Stohr T, Palomo C, Piedra PA, Gilbert BE, Mas V, Millar A, Power UF, Stortelers C, Allosery K, Melero JA, Depla E 2016 Generation and characterization of ALX-0171, a potent novel therapeutic nanobody for the treatment of respiratory syncytial virus infection. Antimicrob Agents Chemother 60:6–13. doi:10.1128/AAC.01802-15. PMC4704182 26438495

[20] DeVincenzo JP, McClure MW, Symons JA, Fathi H, Westland C, Chanda S, Lambkin-Williams R, Smith P, Zhang Q, Beigelman L, Blatt LM, Fry J 2015 Activity of oral ALS-008176 in a respiratory syncytial virus challenge study. N Engl J Med 373:2048–2058. doi:10.1056/NEJMoa1413275. 26580997

[21] Hall CB, Douglas RG Jr, Schnabel KC, Geiman JM 1981 Infectivity of respiratory syncytial virus by various routes of inoculation. Infect Immun 33:779–783. 7287181 10.1128/iai.33.3.779-783.1981 PMC350778

[22] Welliver TP, Garofalo RP, Hosakote Y, Hintz KH, Avendano L, Sanchez K, Velozo L, Jafri H, Chavez-Bueno S, Ogra PL, McKinney L, Reed JL, Welliver RC Sr 2007 Severe human lower respiratory tract illness caused by respiratory syncytial virus and influenza virus is characterized by the absence of pulmonary cytotoxic lymphocyte responses. J Infect Dis 195:1126–1136. doi:10.1086/512615. 17357048 PMC7109876

[23] Johnson JE, Gonzales RA, Olson SJ, Wright PF, Graham BS 2007 The histopathology of fatal untreated human respiratory syncytial virus infection. Mod Pathol 20:108–119. doi:10.1038/modpathol.3800725. 17143259

[24] Sudo K, Miyazaki Y, Kojima N, Kobayashi M, Suzuki H, Shintani M, Shimizu Y 2005 YM-53403, a unique anti-respiratory syncytial virus agent with a novel mechanism of action. Antiviral Res 65:125–131. doi:10.1016/j.antiviral.2004.12.002. 15708639

[25] Noton SL, Nagendra K, Dunn EF, Mawhorter ME, Yu Q, Fearns R 2015 Respiratory syncytial virus inhibitor AZ-27 differentially inhibits different polymerase activities at the promoter. J Virol 89:7786–7798. doi:10.1128/JVI.00530-15. 25995255 PMC4505683

[26] Xiong H, Foulk M, Aschenbrenner L, Fan J, Tiong-Yip CL, Johnson KD, Moustakas D, Fleming PR, Brown DG, Zhang M, Ferguson D, Wu D, Yu Q 2013 Discovery of a potent respiratory syncytial virus RNA polymerase inhibitor. Bioorg Med Chem Lett 23:6789–6793. doi:10.1016/j.bmcl.2013.10.018. 24211022

[27] Liuzzi M, Mason SW, Cartier M, Lawetz C, McCollum RS, Dansereau N, Bolger G, Lapeyre N, Gaudette Y, Lagace L, Massariol MJ, Do F, Whitehead P, Lamarre L, Scouten E, Bordeleau J, Landry S, Rancourt J, Fazal G, Simoneau B 2005 Inhibitors of respiratory syncytial virus replication target cotranscriptional mRNA guanylylation by viral RNA-dependent RNA polymerase. J Virol 79:13105–13115. doi:10.1128/JVI.79.20.13105-13115.2005. 16189012 PMC1235819

[28] Hunt SM, Onions ST, Sherbukhin V, Fordyce EAF, Murray PJ, Brookes DW, Ito K, Strong P 14 4 2016 Novel 5,6-dihydro-4H-benzo[b]thieno-[2,3-d]azepine derivative. Patent WO/2016/055791A1.

[29] Tiong-Yip CL, Aschenbrenner L, Johnson KD, McLaughlin RE, Fan J, Challa S, Xiong H, Yu Q 2014 Characterization of a respiratory syncytial virus L protein inhibitor. Antimicrob Agents Chemother 58:3867–3873. doi:10.1128/AAC.02540-14. 24777090 PMC4068518

[30] Bem RA, Domachowske JB, Rosenberg HF 2011 Animal models of human respiratory syncytial virus disease. Am J Physiol Lung Cell Mol Physiol 301:L148–156. doi:10.1152/ajplung.00065.2011. 21571908 PMC3154630

[31] DeVincenzo JP, Wilkinson T, Vaishnaw A, Cehelsky J, Meyers R, Nochur S, Harrison L, Meeking P, Mann A, Moane E, Oxford J, Pareek R, Moore R, Walsh E, Studholme R, Dorsett P, Alvarez R, Lambkin-Williams R 2010 Viral load drives disease in humans experimentally infected with respiratory syncytial virus. Am J Respir Crit Care Med 182:1305–1314. doi:10.1164/rccm.201002-0221OC. 20622030 PMC3001267

[32] Thompson TM, Roddam PL, Harrison LM, Aitken JA, DeVincenzo JP 2015 Viral specific factors contribute to clinical respiratory syncytial virus disease severity differences in infants. Clin Microbiol 4(3):206. doi:10.4172/2327-5073.1000206. 26473163 PMC4603536

[33] Wu H, Pfarr DS, Johnson S, Brewah YA, Woods RM, Patel NK, White WI, Young JF, Kiener PA 2007 Development of motavizumab, an ultra-potent antibody for the prevention of respiratory syncytial virus infection in the upper and lower respiratory tract. J Mol Biol 368:652–665. doi:10.1016/j.jmb.2007.02.024. 17362988

[34] Zhang L, Peeples ME, Boucher RC, Collins PL, Pickles RJ 2002 Respiratory syncytial virus infection of human airway epithelial cells is polarized, specific to ciliated cells, and without obvious cytopathology. J Virol 76:5654–5666. doi:10.1128/JVI.76.11.5654-5666.2002. 11991994 PMC137037

[35] Villenave R, Shields MD, Power UF 2013 Respiratory syncytial virus interaction with human airway epithelium. Trends Microbiol 21:238–244. doi:10.1016/j.tim.2013.02.004. 23523320

[36] Chapman J, Abbott E, Alber DG, Baxter RC, Bithell SK, Henderson EA, Carter MC, Chambers P, Chubb A, Cockerill GS, Collins PL, Dowdell VC, Keegan SJ, Kelsey RD, Lockyer MJ, Luongo C, Najarro P, Pickles RJ, Simmonds M, Taylor D, Tyms S, Wilson LJ, Powell KL 2007 RSV604, a novel inhibitor of respiratory syncytial virus replication. Antimicrob Agents Chemother 51:3346–3353. doi:10.1128/AAC.00211-07. 17576833 PMC2043207

[37] Deval J, Hong J, Wang G, Taylor J, Smith LK, Fung A, Stevens SK, Liu H, Jin Z, Dyatkina N, Prhavc M, Stoycheva AD, Serebryany V, Liu J, Smith DB, Tam Y, Zhang Q, Moore ML, Fearns R, Chanda SM, Blatt LM, Symons JA, Beigelman L 2015 Molecular basis for the selective inhibition of respiratory syncytial virus RNA polymerase by 2′-fluoro-4′-chloromethyl-cytidine triphosphate. PLoS Pathog 11:e1004995. doi:10.1371/journal.ppat.1004995. 26098424 PMC4476725

[38] Perron M, Stray K, Kinkade A, Theodore D, Lee G, Eisenberg E, Sangi M, Gilbert BE, Jordan R, Piedra PA, Toms GL, Mackman R, Cihlar T 2015 GS-5806 inhibits a broad range of respiratory syncytial virus clinical isolates by blocking the virus-cell fusion process. Antimicrob Agents Chemother 60:1264–1273. doi:10.1128/AAC.01497-15. 26666922 PMC4776015

[39] Deval J, Fung A, Stevens SK, Jordan PC, Gromova T, Taylor JS, Hong J, Meng J, Wang G, Dyatkina N, Prhavc M, Symons JA, Beigelman L 2016 Biochemical effect of resistance mutations against synergistic inhibitors of RSV RNA polymerase. PLoS One 11:e0154097. doi:10.1371/journal.pone.0154097. 27163448 PMC4862670

[40] Eltahla AA, Luciani F, White PA, Lloyd AR, Bull RA 2015 Inhibitors of the hepatitis C virus polymerase; mode of action and resistance. Viruses 7:5206–5224. doi:10.3390/v7102868. 26426038 PMC4632376

[41] Christen MT, Menon L, Myshakina NS, Ahn J, Parniak MA, Ishima R 2012 Structural basis of the allosteric inhibitor interaction on the HIV-1 reverse transcriptase RNase H domain. Chem Biol Drug Des 80:706–716. doi:10.1111/cbdd.12010. 22846652 PMC3465473

[42] Ghildyal R, Ho A, Jans DA 2006 Central role of the respiratory syncytial virus matrix protein in infection. FEMS Microbiol Rev 30:692–705. doi:10.1111/j.1574-6976.2006.00025.x. 16911040

[43] Do LA, Wilm A, Van Doorn HR, Lam HM, Sim S, Sukumaran R, Tran AT, Nguyen BH, Tran TT, Tran QH, Vo QB, Dac NA, Trinh HN, Nguyen TT, Binh BT, Le K, Nguyen MT, Thai QT, Vo TV, Ngo NQ, Dang TK, Cao NH, Tran TV, Ho LV, Farrar J, Jong M, Chen S, Nagarajan N, Bryant JE, Hibberd ML 2015 Direct whole-genome deep-sequencing of human respiratory syncytial virus A and B from Vietnamese children identifies distinct patterns of inter- and intra-host evolution. J Gen Virol 96:3470–3483. doi:10.1099/jgv.0.000298. 26407694 PMC4804761

[44] Grad YH, Newman R, Zody M, Yang X, Murphy R, Qu J, Malboeuf CM, Levin JZ, Lipsitch M, DeVincenzo J 2014 Within-host whole-genome deep sequencing and diversity analysis of human respiratory syncytial virus infection reveals dynamics of genomic diversity in the absence and presence of immune pressure. J Virol 88:7286–7293. doi:10.1128/JVI.00038-14. 24741088 PMC4054443

[45] Mackman RL, Sangi M, Sperandio D, Parrish JP, Eisenberg E, Perron M, Hui H, Zhang L, Siegel D, Yang H, Saunders O, Boojamra C, Lee G, Samuel D, Babaoglu K, Carey A, Gilbert BE, Piedra PA, Strickley R, Iwata Q, Hayes J, Stray K, Kinkade A, Theodore D, Jordan R, Desai M, Cihlar T 2015 Discovery of an oral respiratory syncytial virus (RSV) fusion inhibitor (GS-5806) and clinical proof of concept in a human RSV challenge study. J Med Chem 58:1630–1643. doi:10.1021/jm5017768. 25574686

[46] Wang G, Deval J, Hong J, Dyatkina N, Prhavc M, Taylor J, Fung A, Jin Z, Stevens SK, Serebryany V, Liu J, Zhang Q, Tam Y, Chanda SM, Smith DB, Symons JA, Blatt LM, Beigelman L 2015 Discovery of 4′-chloromethyl-2′-deoxy-3′,5′-di-O-isobutyryl-2′-fluorocytidine (ALS-8176), a first-in-class RSV polymerase inhibitor for treatment of human respiratory syncytial virus infection. J Med Chem 58:1862–1878. doi:10.1021/jm5017279. 25667954

[47] Dochow M, Krumm SA, Crowe JE Jr, Moore ML, Plemper RK 2012 Independent structural domains in paramyxovirus polymerase protein. J Biol Chem 287:6878–6891. doi:10.1074/jbc.M111.325258. 22215662 PMC3307299

[48] Perkins SM, Webb DL, Torrance SA, El Saleeby C, Harrison LM, Aitken JA, Patel A, DeVincenzo JP 2005 Comparison of a real-time reverse transcriptase PCR assay and a culture technique for quantitative assessment of viral load in children naturally infected with respiratory syncytial virus. J Clin Microbiol 43:2356–2362. doi:10.1128/JCM.43.5.2356-2362.2005. 15872266 PMC1153767

[49] Mason SW, Lawetz C, Gaudette Y, Do F, Scouten E, Lagace L, Simoneau B, Liuzzi M 2004 Polyadenylation-dependent screening assay for respiratory syncytial virus RNA transcriptase activity and identification of an inhibitor. Nucleic Acids Res 32:4758–4767. doi:10.1093/nar/gkh809. 15356293 PMC519107

