# Supplementary material for: Preclinical Characterization of PC786, an Inhaled Small-Molecule Respiratory Syncytial Virus L Protein Polymerase Inhibitor
Source: Antimicrob Agents Chemother. 2017 Aug 24;61(9):e00737-17. doi: 10.1128/AAC.00737-17 (PMC5571287; doi:10.1128/AAC.00737-17)
Supplement: Supplemental material [file supp_61_9_e00737-17__index.html]

Supplemental material 

# Preclinical Characterization of PC786, an Inhaled Small-Molecule Respiratory Syncytial Virus L Protein Polymerase Inhibitor

## Supplemental material

- Supplemental file 1 -

  Fig. S1 to S5 and Tables S1 to S4

  PDF, 967K
