# Supplementary material for: Preclinical Characterization of PC786, an Inhaled Small-Molecule Respiratory Syncytial Virus L Protein Polymerase Inhibitor
Source: Antimicrob Agents Chemother. 2017 Aug 24;61(9):e00737-17. doi: 10.1128/AAC.00737-17 (PMC5571287; doi:10.1128/AAC.00737-17)
Supplement: Supplemental material [file AAC.00737-17_zac009176480s1.pdf]

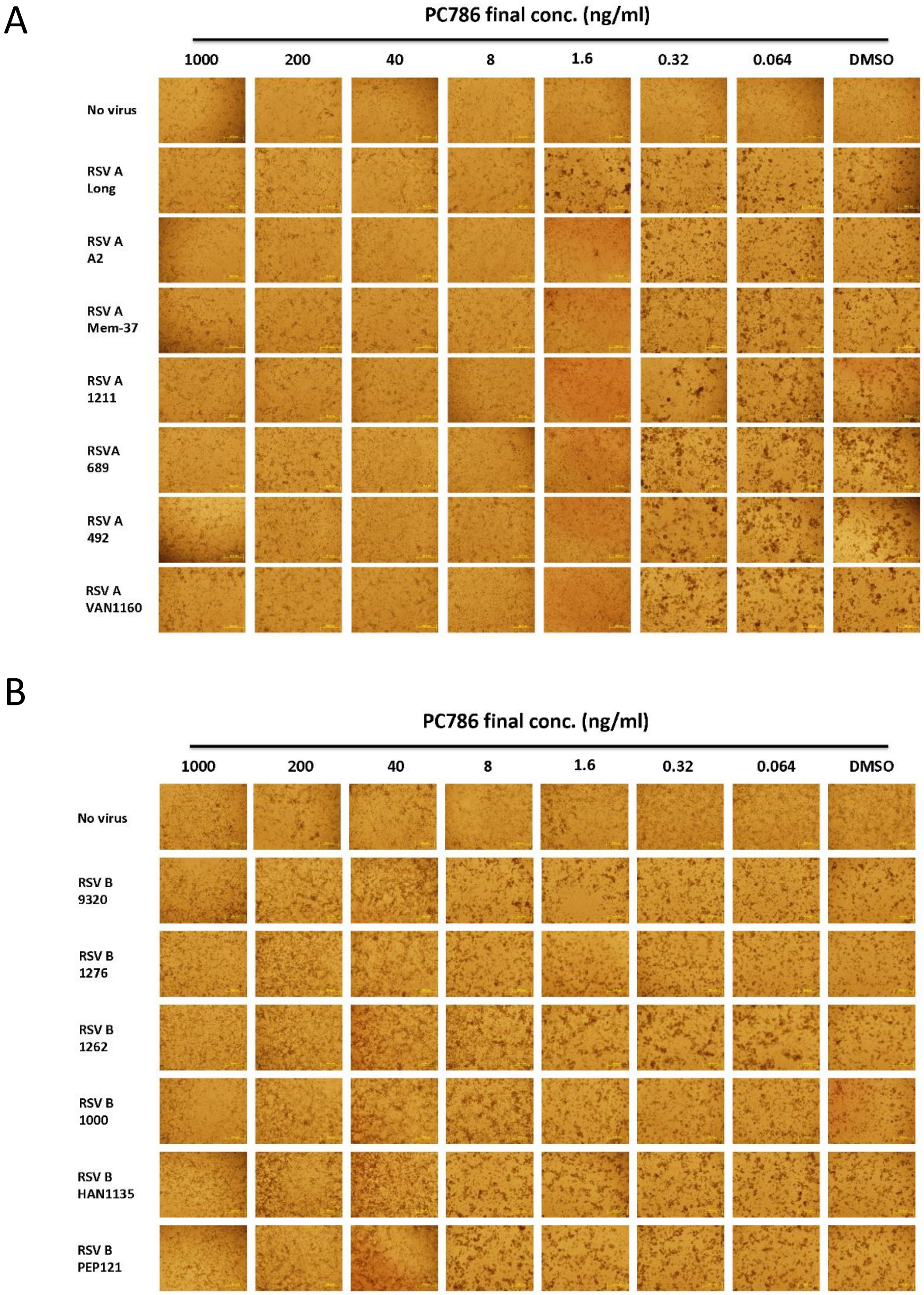

Figure S1 Microscopic image of low passage RSV clinical isolates (panel A: RSV A and Panel B: RSV B)-induced CPE in HEp-2 cells: Effects of PC786

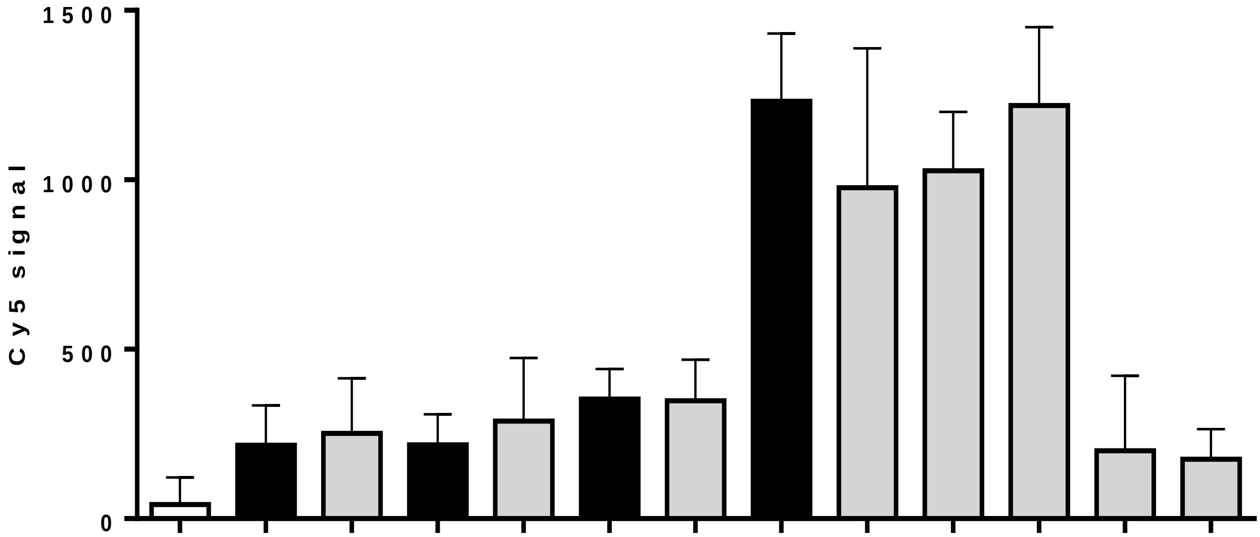

HEp2 cell extracts	-	+	+	+	+	+	+	+	+	+	+	+	+
Vaccinia virus	-	-	-	+	+	+	+	+	+	+	+	+	+
RSV P plasmid	-	-	-	-	-	+	+	+	+	+	+	+	+
RSV **L** plasmid	-	-	-	-	-	-	-	+	+	+	+	+	+
PC786 (nM)	-	-	100	-	100	-	100	-	0.01	0.1	1	10	100

Figure S2

Effects of PC786 on negative control and RSV-L protein associated signal in RdRp activity. HEp-2 cells were uninfected, infected with vaccinia virus (T7 donor), infected with vaccinia virus and then transfected with RSV P protein plasmid only, or infected with vaccinia virus and then transfected with RSV P and L protein plasmid in HEp2 cells. Crude cell extracts were prepared 36hrs after transfection. PC786 was added to reaction including cell extracts, primers and Cy5 ATP and CTP, and incubated for 2hr at 30°C.

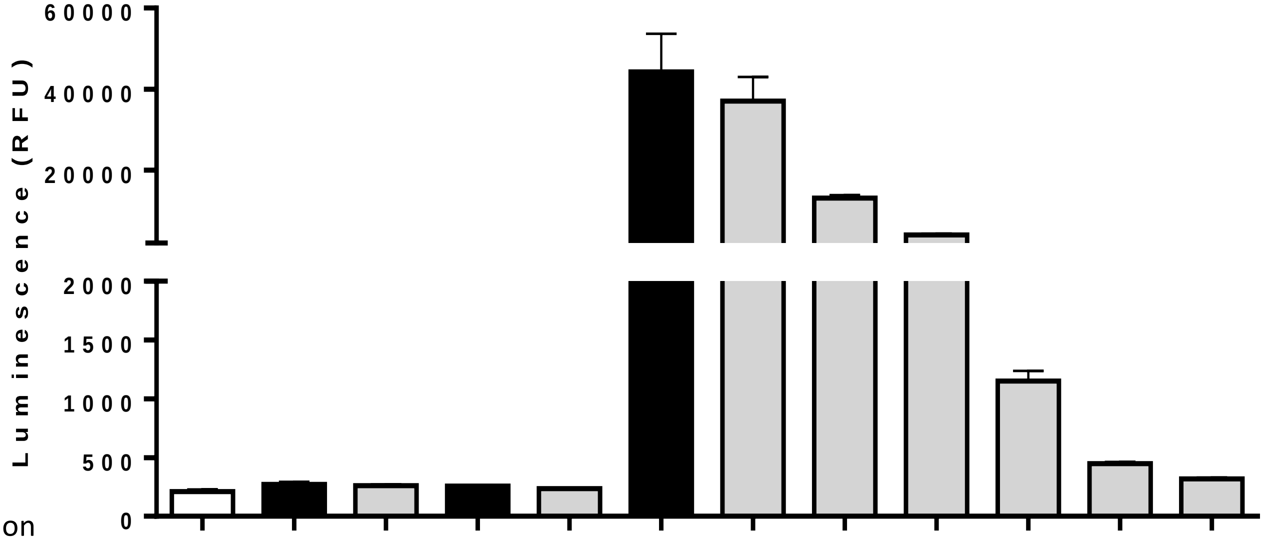

Transfection

Vaccinia virus	-	+	+	+	+	+	+	+	+	+	+	+
RSV P, M2-1, N	-	-	-	+	+	+	+	+	+	+	+	+
**RSV L**	-	-	-	-	-	+	+	+	+	+	+	+
PC786 (nM)	-	-	**140**	-	**140**	-	**0.05**	**0.22**	**1.1**	**5.6**	**28**	**140**

Figure S3

Effects of PC786 on negative control and RSV-L protein associated signal in minigenome analysis. HEp-2 cells were uninfected, infected with vaccinia virus (T7 donor), infected with vaccinia virus and then transfected with RSV P, M2-1 and N protein plasmid only, or infected with vaccinia virus and then transfected with RSV P, M2-1, N and L protein plasmid. PC786 was added 24hrs after infection/transfection, and luminescent signal was measured 48hrs after treatment.

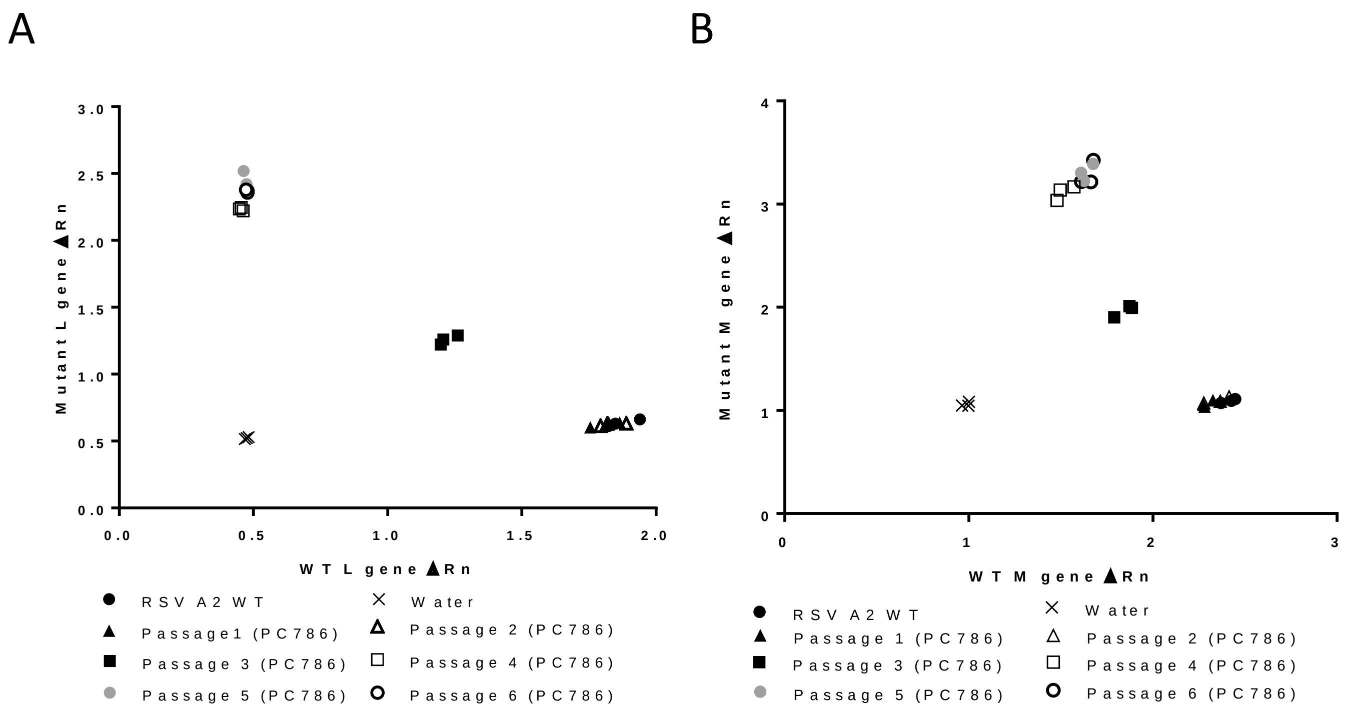

Figure S4

Passage dependent mutation induction on L protein (A) and M protein (B). HEp-2 cells were infected with RSV A2 and passaged in the presence of escalating concentrations of PC786 (1.4nM, 7.0nM, 35nM, 175nM, 873nM and 4366nM from passage 1 to 6, Table S3). The incidence of mutation was determined by SNP analysis.

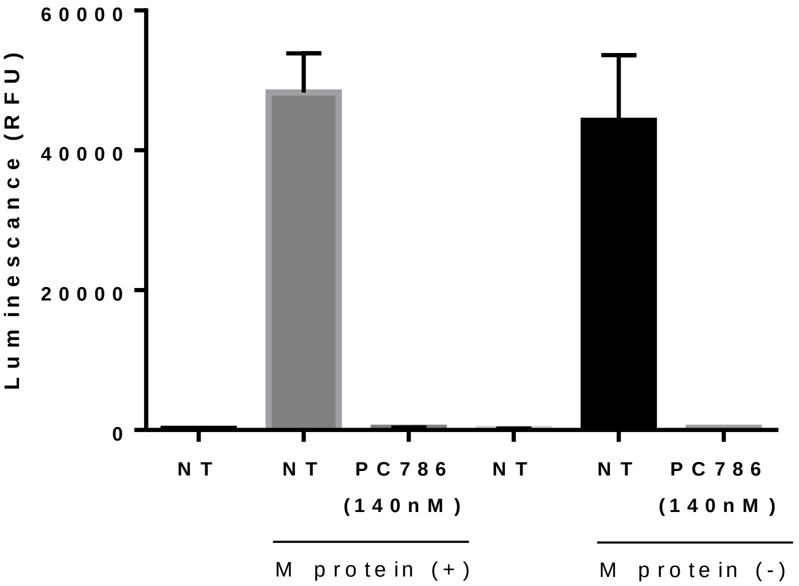

Figure S5

An impact of RSV M protein on minigenome analysis. RSV M protein plasmid was transfected together with L, P, M2-1, N plasmids. Compounds were treated 24hrs after transfection, and the signal was determined 48hrs after treatment.

Table S1 **Effects of PC786 on the CPE induced in HEp-2 cells by low passage RSV clinical isolates**

Compound (Virus strain)	Strain source	PC786 Inhibitory Effects (nM) IC_{50}	IC_{90}
RSVA long	Laboratory	2.46	3.25
RSVA2	Laboratory	0.96	1.82
RSVA Memphis 37c	Clinical	0.22	3.77
RSVA 1211	Clinical	<0.09	1.96
RSVA 689	Clinical	0.50	0.60
RSVA 492	Clinical	0.42	0.57
RSVA VAN1160	Clinical	0.71	1.93
RSVB 9320	Laboratory	29.1	64.3
RSVB 1276	Clinical	50.6*	821*
RSVB 1262	Clinical	14.5	692
RSVB 1000	Clinical	13.4*	342*
RSVB HAN1135	Clinical	25.8	27.4
RSVB PEP121	Clinical	17.5*	196*

CPE=cytopathic effects; IC_{50}/IC_{90}=concentration required for 50/90% inhibition; N=number; ND=not done; RSV=respiratory syncytial virus.
CPE resazurin detection in 384 well format, PC786 and GS-5806 were treated simultaneously with virus infection. * 3 parameter curve fit is used as concentration response does not fit to 4 parameter robust fit analysis

Table S2 Effects of PC786 on RSV titre in supernatant from HEp-2 cells

**Compound (Virus strain)**	**Strain source**	**PC786 Inhibitory Effects (nM)**	
		**IC_{50} 0.3 Log PFUe reduction**	**IC_{90} 1 Log PFUe reduction**
RSVA long	Laboratory	2.65	3.45
RSVA2	Laboratory	1.52	1.69
RSVA Memphis 37c	Clinical	0.53	1.16
RSVA 1211	Clinical	<0.09	1.05
RSVA 689	Clinical	0.54	1.12
RSVA 492	Clinical	0.43	0.50
RSVA VAN1160	Clinical	0.64	0.91
RSVB 9320	Laboratory	30.3	31.0
RSVB 1276	Clinical	15.1	22.5
RSVB 1262	Clinical	9.36	12.3
RSVB 1000	Clinical	10.3	12.4
RSVB HAN1135	Clinical	8.1	34.0
RSVB PEP121	Clinical	17.7	27.4

CPE=cytopathic effects; IC_{50}/IC_{90}=concentration required for 50/90% inhibition; N=number; ND=not done; RSV=respiratory syncytial virus.
CPE resazurin detection in 384 well format, PC786 and GS-5806 were treated simultaneously with virus infection. * 3 parameter curve fit is used as concentration response does not fit to 4 parameter robust fit analysis

Table S3

Treatment regimen of the used for mutation induction

Passage Number	Concentration of PC786 (nM)
1	1.4
2	7.0
3	35
4	175
5	873
6	4366

Table S4 Inhibition of wild type virus and PC786 escape mutant induced CPE by antiviral compounds

Compounds (N=3)	Wild type RSVA2		PC786 escape mutant	
	IC_{50} (nM)	IC_{90} (nM)	IC_{50} (nM)	IC_{90} (nM)
PC786	0.027	0.065	17.9	1120
BI Compound D	11.9	79.9	87.4	161
AZ compound 11J	0.023	0.089	>1580	>1580
GS-5806	0.0018	0.0048	<0.188	<0.188
